# Atypical Hydrogen Bond Interaction Enables Anion‐Rich Solvation Structure in Polymer Electrolytes for High‐Voltage Flexible Lithium Metal Batteries

**DOI:** 10.1002/advs.202507007

**Published:** 2025-06-11

**Authors:** Shujing Wen, Junhua Zhou, Guangzhao Zhang, Qingrong Wang, Chao Luo, Ruo Wang, Pengxian Li, Chaoyang Wang, Xiaoxiong Xu, Yonghong Deng, Jian Chang, Zijian Zheng

**Affiliations:** ^1^ Department of Applied Biology and Chemical Technology Faculty of Science The Hong Kong Polytechnic University Hung Hom Hong Kong SAR 999077 China; ^2^ Department of Materials Science and Engineering, School of Innovation and Entrepreneurship Key University Laboratory of Highly Efficient Utilization of Solar Energy and Sustainable Development of Guangdong Southern University of Science and Technology Shenzhen 518055 China; ^3^ Dongguan Key Laboratory of Interdisciplinary Science for Advanced Materials and Large‐Scale Scientific Facilities School of Physical Sciences Great Bay University Dongguan Guangdong 523000 China; ^4^ Research Institute of Materials Science South China University of Technology Guangzhou 510640 China; ^5^ Research Institute for Smart Energy The Hong Kong Polytechnic University Hung Hom Hong Kong SAR 999077 China; ^6^ Research Institute for Intelligent Wearable Systems The Hong Kong Polytechnic University Hung Hom Hong Kong SAR 999077 China; ^7^ PolyU‐Daya Bay Technology and Innovation Research Institute Huizhou Guangdong 516083 China; ^8^ PolyU‐Wenzhou Technology and Innovation Research Institute Wenzhou Zhejiang 325024 China

**Keywords:** atypical hydrogen bond, high‐voltage lithium metal battery, interphase, polymer solid electrolyte, solvation structure

## Abstract

Flexible lithium metal batteries (LMBs) using polymer‐based solid‐state electrolytes (PSSEs) are highly desirable for wearable applications because of the potential advantages in energy density and safety. Recently, ether‐based polyelectrolytes have received extensive attention because of their good stability, high ionic conductivity, and Li metal anode compatibility. However, it typically forms organic‐rich cathode electrolyte interphase (CEI) at the cathode, which is still a pain point that impedes the high‐voltage performance. To address this challenge, herein a fluorinated plasticizer, bis(2‐fluoroethyl) ether (BFE) is reported, which can be easily blended into ether‐based PSSE and enables high‐voltage‐stable flexible LMBs. The BFE and PSSE molecules form an atypical hydrogen bond interaction, which weakens the interaction between PSSE and lithium ions. This leads to the formation of an anion‐rich solvation structure that generates inorganic‐rich and high‐voltage‐stable CEI. The oxidation stability of PSSE is improved from 4.4 V to over 4.7 V after introducing the BFE molecules. LMBs using BFE‐blended PSSE can couple with high‐voltage cathode and retain 80% capacity after 480 cycles at 1C. Full cells show high energy density (752.2 Wh L^−1^) outstanding capacity retention per cycle (99.88%), and high flexibility with almost identic charge/discharge characteristics after 4000 bending cycles.

## Graphical abstract

A high‐voltage, Li‐metal‐compatible, and highly conductive polymer electrolyte is reported by integrating a low‐solvating fluorinated plasticizer. Strong atypical hydrogen bonds formed between the plasticizer and polymer chains promote the establishment of anion‐rich solvation structure, effectively stabilizing electrolyte‐electrode interphase at the anode and cathode side. High‐voltage solid‐state Li metal full cells show a high volumetric energy density, outstanding capacity retention, and desirable flexibility.

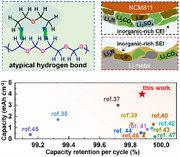

## 1 Introduction

Flexible lithium (Li) metal battery (LMB) provides a promising technology toward high‐energy‐density energy storage in the near future.^[^ ^1^, ^2^ ^]^ This is particularly interesting to wearable and soft electronic applications where high energy density is among the top priorities. However, the easy denitrification of Li metal anodes occurring in conventional liquid electrolytes causes rapid performance decay and cell failure of LMBs.^[^ ^3^, ^4^, ^5^ ^]^ The use of liquid electrolytes also imposes a serious safety concern on the use of Li metals.^[^ ^6^, ^7^ ^]^ Therefore, the research communities and industries have been working extensively in recent years to develop solid‐state electrolytes with high mechanical strength, stability, and conductivity to replace liquid electrolytes.^[^ ^8^, ^9^, ^10^, ^11^, ^12^ ^]^ In particular, polymer‐based solid‐state electrolytes (PSSEs) are of great interest because of their remarkable processability and better interfacial contact with electrodes, in comparison to inorganic types of solid‐state electrolytes.^[^ ^13^, ^14^, ^15^, ^16^, ^17^, ^18^, ^19^ ^]^

Ether‐based polyelectrolyte, e.g., poly(1,3‐dioxolane) (PDOL), is an emerging high‐performance PSSE.^[^ ^20^, ^21^ ^]^ Its ionic conductivity is relatively high (>10^−5^ S cm^−1^) among the reported PSSEs, because the flexible ether‐oxygen group can rapidly conduct Li^+^.^[^ ^22^ ^]^ Furthermore, ether‐based polyelectrolytes are highly compatible with Li metal anode because of its weak solvation ability and the formation of stable solid electrolyte interphase (SEI).^[^ ^23^ ^]^ More importantly, using Li salts with Lewis acidity (e.g., lithium hexafluorophosphate, LiPF_6_; lithium tetrafluoroborate, LiBF_4_; and lithium bis(fluorosulfonyl)imide, LiFSI),^[^ ^24^, ^25^, ^26^ ^]^ some ether‐based polyelectrolytes can be fabricated through in situ polymerization in the battery cell with preloaded precursor solution. This is a remarkable advantage over many other types of PSSEs, where Li‐reacting initiators such as 2,2'‐Azobis(2‐methylpropionitrile) are required in the polymerization.^[^ ^27^, ^28^ ^]^ The in situ polymerization not only offers a rapid fabrication method but also enables the gapless contact between the polyelectrolyte and the electrodes, resulting in low interphase impedance and enhanced battery performance.^[^ ^29^ ^]^ Nonetheless, ether‐based polyelectrolyte shows poor tolerance to high‐voltage operation due to the generation of unstable organic‐rich cathode electrolyte interphase (CEI), which are susceptible to be oxidized at high voltage.^[^ ^30^ ^]^ For example, the operation voltage of PDOL is normally under 4 V (vs. Li/Li^+^), which significantly hampers its application with high‐voltage cathode materials (e.g., lithium nickel manganese cobalt oxides, NCM) and hence limits the energy density of LMBs.^[^ ^15^ ^]^

Several strategies have been reported to increase the operation voltage of ether‐based polyelectrolytes. Introducing inorganic nanoparticles such as yttria‐stabilized zirconia,^[^ ^25^ ^]^ silicon dioxide,^[^ ^31^ ^]^ and lanthanum titanate lithium^[^ ^32^ ^]^ into PDOL can increase the oxidation resistance, but these metal oxides will react with Li metal and cause low Coulombic efficiency (CE). It is also effective by applying high‐concentration Li salts to tune the solvation structure of the electrolyte to form a stable inorganic‐rich interphase. However, this method is costly and will decrease the ionic conductivity of the polyelectrolyte.^[^ ^24^ ^]^ In the third strategy, liquid plasticizers such as fluoroethylene carbonate,^[^ ^33^ ^]^ ethylene carbonate,^[^ ^34^ ^]^ vinylene carbonate,^[^ ^35^ ^]^ succinonitrile,^[^ ^36^ ^]^ and ionic liquids,^[^ ^37^ ^]^ can enhance the oxidation stability by generating stable CEI, but these high‐solvating solvents will damage Li metal efficiency sharply. Therefore, it is crucial to develop a strategy that can improve the high‐voltage stability of ether‐based PSSE without sacrificing its high ionic conductivity and good compatibility with Li metal anode.

**Figure 1:**
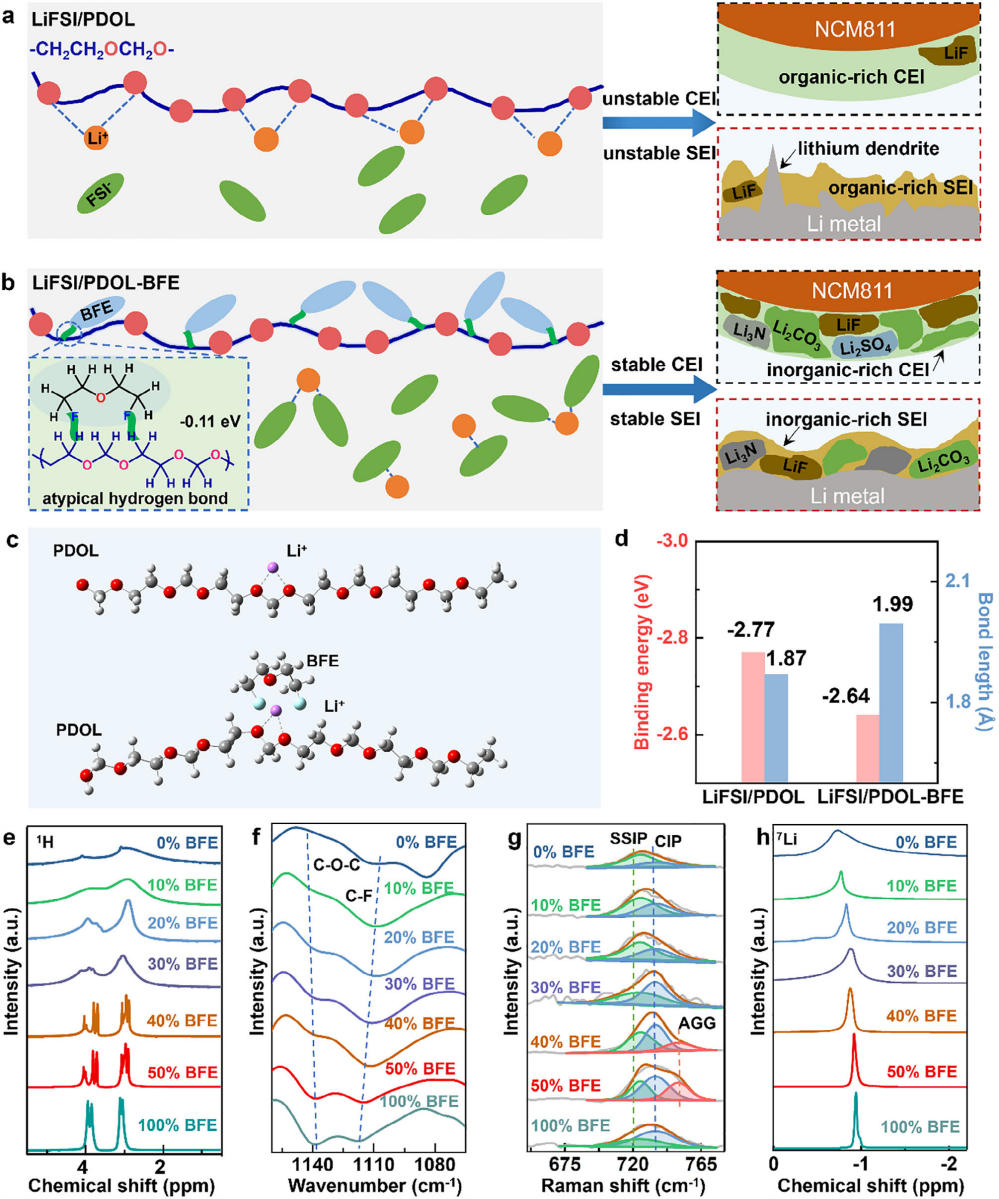
Design principle and solvation structure. Schematic illustrating the solvation structure and interphase information in PDOL a) and PDOL‐BFE b). c) Optimized geometric configurations showing interactions between Li^+^ and PDOL or PDOL‐BFE. d) Binding energies and bond lengths between Li^+^ and PDOL or PDOL‐BFE. ^1^H NMR e), FTIR f), Raman g), and ^7^Li NMR h) spectra of PDOL‐BFE with various concentrations of BFE. PDOL, 10%BFE, 20%BFE, 30%BFE, 40%BFE, 50%BFE, and BFE represent 2.0 m LiFSI in DOL, 2.0 m LiFSI in DOL‐BFE (10 vol% BFE), 2.0 m LiFSI in DOL‐BFE (20 vol% BFE), 2.0 m LiFSI in DOL‐BFE (30 vol% BFE), 2.0 m LiFSI in DOL‐BFE (40 vol% BFE), 2.0 m LiFSI in DOL‐BFE (50 vol% BFE), and 2.0 m LiFSI in BFE (100 vol% BFE) respectively.

Herein, we report a high‐voltage, Li‐metal‐compatible, and highly conductive ether‐based PSSE via blending a low‐solvating fluorinated plasticizer, bis(2‐fluoroethyl) ether (BFE) with PDOL. Strong atypical hydrogen bonds can be generated between the F atoms in BFE and the H atoms in PDOL (**Figure** **1**a,b). The interaction will weaken the coordination between PDOL chain and Li^+^, and form an anion‐rich solvation structure, which facilitates the generation of stable CEI and SEI. Consequently, the electrolyte shows ultrahigh oxidation stability of more than 4.7 V, along with a high Li metal CE of 98.5%, which outperforms reported PSSEs tested under similar conditions. The liquid BFE with certain solvation ability also contributes to the high Li^+^ conductivity of the electrolyte (5.0 × 10^−4^ S cm^−1^). Solid‐state LMBs coupling with high‐voltage NCM_811_ cathode retain 80% capacity after 480 cycles under high rate cycling at 1C. Full cells show a high volumetric energy density of 752.2 Wh L^−1^ and outstanding capacity retention per cycle (99.88%). To the best of our knowledge, this cycling stability is superior to all other reported PSSE‐based LMBs. Furthermore, the electrolyte endows pouch cells with high flexibility, showing almost identical capacity utilization after 4000 bending cycles.

## 2 Results and Discussion

### 2.1 Anion‐Rich Solvation Structure Enabled by Atypical Hydrogen Bonds

The intermolecular force between PDOL and BFE (C─H─F) is stronger than typical van der Waals forces but weaker than covalent bonds and hydrogen bonds (e.g., O─H─F), and is considered as atypical hydrogen bond.^[^ ^38^ ^]^ The unique interaction is first revealed by density functional theory (DFT) calculations with a negative binding energy of ‐0.11 eV between PDOL and BFE (Figure S1, Supporting Information). Moreover, the binding energy between Li^+^ and PDOL segments decreases from −2.77 to −2.64 eV with the addition of BFE (Figure 1c,d), accompanied by an elongation of the Li^+^─O bond length from 1.87 to 1.99 Å, suggesting weakened coordination between Li⁺ and PDOL.

The existence of atypical hydrogen bond interaction between PDOL and BFE is further approved by Nuclear magnetic resonance (NMR) and Fourier transform infrared (FTIR) spectroscopy. The LiFSI/PDOL‐BFE PSSE was prepared through in situ polymerization of DOL, initiated by lithium bisfluorosulfonimide (LiFSI) (Figures S2 and S3, Supporting Information). As shown in Figure 1c, the weak signal of ^1^H NMR peaks in the LiFSI/PDOL (0% BFE) demonstrates the polymerization of DOL. As the ratio of BFE increases, the ^1^H NMR peaks shift downfield. This is attributed to the high electronegativity of the F atom in BFE, which withdraws the electron cloud of H atoms in PDOL through inductive effects, resulting in the deshielding effect of ^1^H nuclei and consequent downfield displacement. Both the downfield shift of ^1^H NMR peaks and the upfield shift of ^19^F NMR peak (Figure S4, Supporting Information) prove the existence of an atypical hydrogen bond between BFE and PDOL. In FTIR spectra, PDOL and BFE display the characteristic C─O─C and C─F bond, respectively (Figure 1d). When the ratio of BFE increases from 10% to 50%, the C─F peak shifts from 1108 to 1114 cm^−1^. This blueshift suggests the contraction of C─F bonds, indicating an increase in the electron cloud density of the F atom. This further implies the generation of the atypical hydrogen bond between PDOL and BFE (C─H···F─C).

Due to the formation of atypical hydrogen bonds, the BFE weakens the coordination between PDOL and Li^+^ ion, forming an anion‐rich solvation structure. Typically, there are three kinds of Li^+^ ion coordination environments, including solvent‐separated ion pair (SSIP, solvent‐surrounded Li^+^), contact ion pair (CIP, Li^+^‐anion single pair), and ion aggregates (AGG, Li^+^‐anion cluster). With the increase of BFE, Raman spectra show a blue shift, ascribed to the gradual transformation of Li^+^ ion coordination from SSIP‐dominated (725 cm^−1^ at 10% BFE), to CIP‐dominated (735 cm^−1^ at 30% BFE), and finally to AGG‐dominated (750 cm^−1^ at 50% BFE) structures (Figure 1e; Figure S5, Supporting Information). The result demonstrates that the strong atypical hydrogen bond interaction between PDOL and BFE increases the amount of AGG in LiFSI/PDOL‐BFE and generates an anion‐rich solvation structure. As the ratio of BFE increases, the C─O─C peak shows a redshift from 1145 cm^−^¹ in 10% BFE to 1137 cm^−^¹ in 50% BFE based on the FTIR spectra (Figure 1d). The redshift means the stretched C─O─C bond, which implies the decreased coordination between ether oxygen atoms on PDOL and Li^+^. In ^7^Li NMR spectra, as the ratio of BFE increases, the peak shifts upfield from −0.77 ppm in 10% BFE to −0.92 ppm in 50% BFE. This indicates a preferential Li^+^ coordination with FSI^−^ anions over PDOL chains, consistent with the results mentioned above.

### 2.2 Compositions and Structures of CEI and SEI

**Figure 2:**
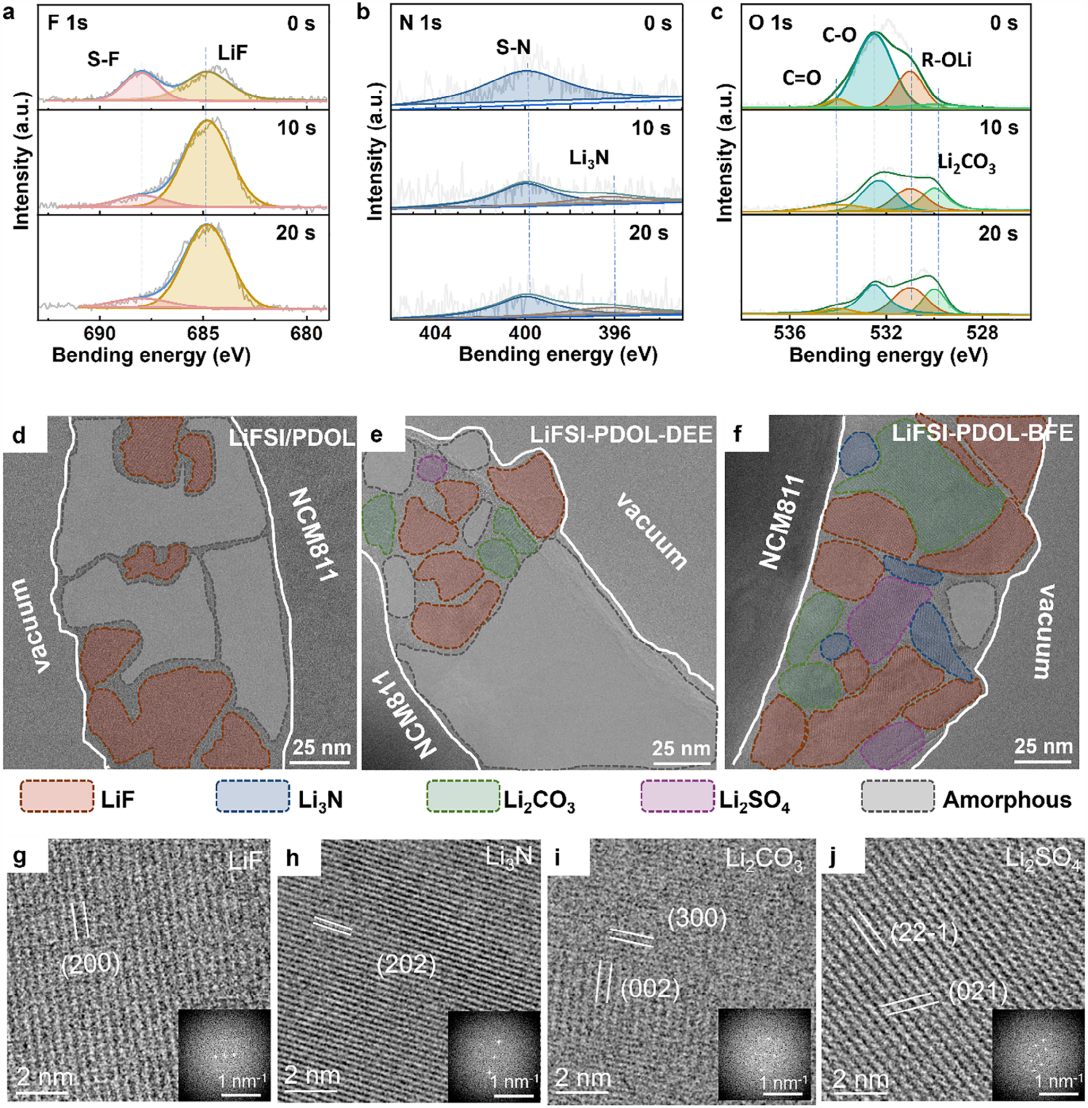
CEI composition and structure formed on the surface of the NCM_811_ cathode in various electrolytes after 50 cycles. a–c) F 1s, N 1s, and O 1s XPS spectra with various etching times in LiFSI/PDOL‐BFE. d–f) Cryo‐TEM images of CEI in various electrolytes. g–j) High‐resolution Cryo‐TEM images and corresponding FFT patterns of inorganic CEI species in LiFSI/PDOL‐BFE.

The anion‐dominated solvation structure in LiFSI/PDOL‐BFE can facilitate generating inorganic‐rich CEI. The LiFSI/PDOL‐diethyl ether (DEE) PSSE was also prepared as a comparison, where the DEE is an ether solvent with an identical structure to that of BFE except for the absence of F atoms. X‐ray photoelectron spectroscopy (XPS) depth profiling of cycled cathode in various electrolytes reveals the 3D composition of CEI. In C 1s XPS spectra (Figures S6c,–f, and S7a, Supporting Information), four kinds of C species are shown in all electrolytes, including C─C (284.8 eV), C─O (285.5 eV), C═O (286.8 eV), and C─F (290.3 eV). Compared to LiFSI/PDOL‐DEE and LiFSI/PDOL‐BFE, the CEI in LiFSI/PDOL contains significantly higher amounts of organic C─O and C═O components, attributed to the PDOL polymer matrix decomposition. In F 1s XPS spectra (**Figure** **2**a; Figure S6a–d, Supporting Information), both organic S‐F (688 eV) and inorganic lithium fluoride (LiF, 684.8 eV) species are shown in all electrolytes, originating from the decomposition of FSI^−^ anions. Notably, LiFSI/PDOL‐BFE exhibits a substantially higher LiF content than LiFSI/PDOL and LiFSI/PDOL‐DEE, particularly after 20 s of etching. The N 1s spectra (Figure 2b) further confirm a higher concentration of inorganic lithium nitride (Li_3_N, 396.1 eV) in LiFSI/PDOL‐BFE compared to the other electrolytes. This trend is reinforced by the O 1s and S 2p spectra (Figure 2c; Figure S7b,c, Supporting Information), which reveal greater amounts of inorganic lithium carbonate (Li_2_CO_3_, 530.5 eV in O 1s) and lithium sulfate (Li_2_SO_4_, 67.9 eV in S 2p_3/2_) in LiFSI/PDOL‐BFE, further supporting the formation of an inorganic‐rich CEI.

The morphology and structure of CEI are explored by cryo‐transmission electron microscope (Cryo‐TEM). In LiFSI/PDOL, moderate amounts of inorganic nanocrystallines with non‐continuous distribution are embedded in an amorphous organic matrix (Figure 2d). High‐resolution Cryo‐TEM and corresponding fast Fourier transform (FFT) images reveal that these nanocrystallines are LiF with characteristic (200) lattice plane (Figure S8a, Supporting Information). Although LiF, Li_2_CO_3_ with (202) lattice plane, and Li_2_SO_4_ with (222) and (311) lattice plane all appear in LiFSI/PDOL‐DEE (Figure 2e; Figure S8b–d, Supporting Information), the content of these inorganic species is insufficient and their distribution is also non‐uniform. In contrast, the CEI in LiFSI/PDOL‐BFE exhibits a dense and uniform distribution of inorganic species, including LiF, Li_2_CO_3_, Li_2_SO_4_, and Li_3_N (with the (202) lattice plane) (Figure 2f–j). Electron energy loss spectroscopy (EELS) mapping images (Figure S9, Supporting Information) further display the elemental distribution in CEI. An obvious F signal is shown on the surface of the cathode in LiFSI/PDOL‐BFE, demonstrating its F‐rich CEI nature.

**Figure 3:**
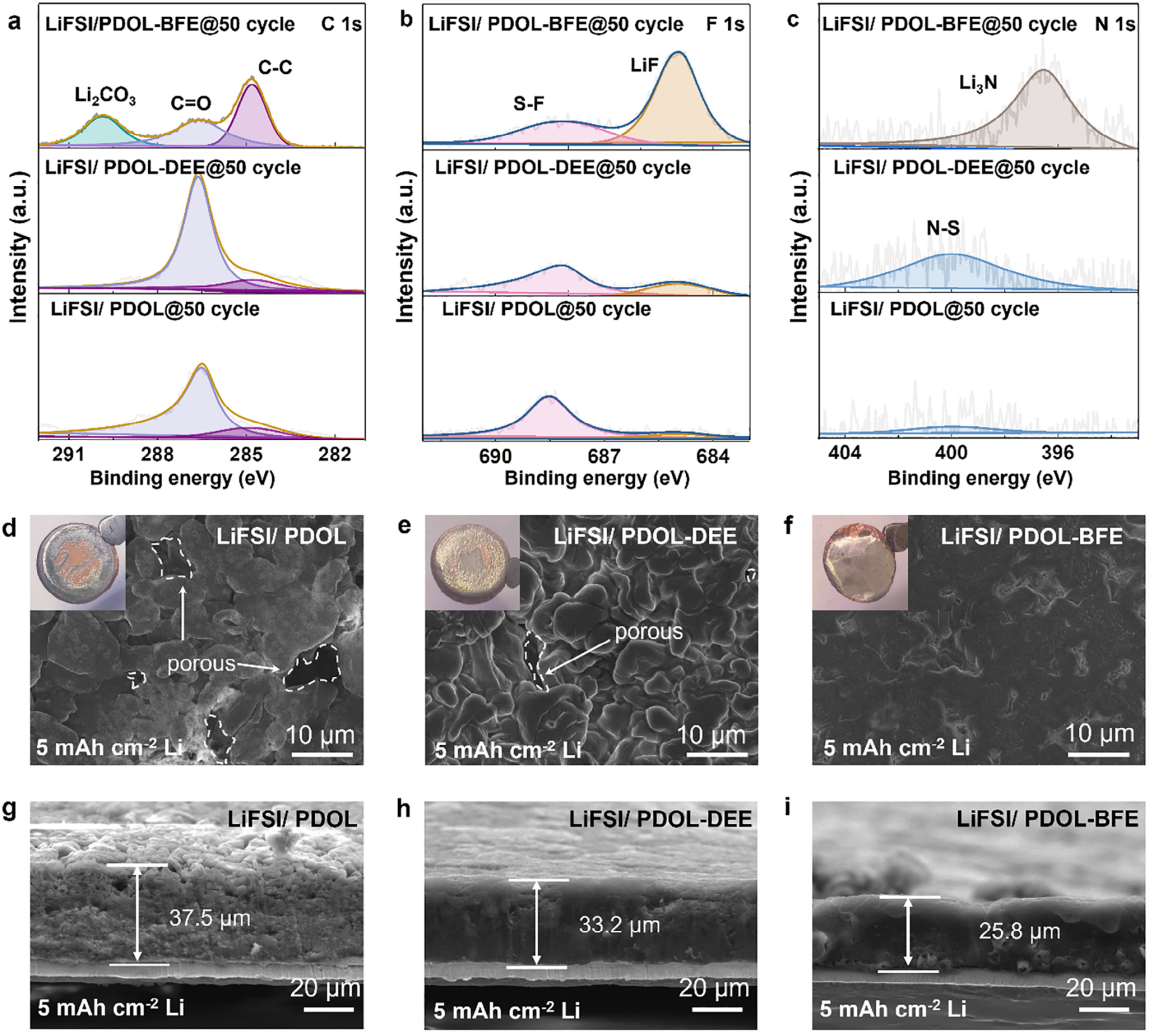
SEI composition and Li metal morphology in various electrolytes. a–c) C 1s, F 1s, and N 1s XPS spectra of Li metal after 50 cycles. Top‐view d–f) and corresponding cross‐sectional g–i) SEM images of deposited 5 mAh cm^−2^ lithium metal.

The anion‐dominated solvation structure in LiFSI/PDOL‐BFE also facilitates generating inorganic‐rich SEI at Li metal anode side. C1s XPS spectra (**Figure** **3**a) reveal that only organic species are shown in LiFSI/PDOL and LiFSI/PDOL‐DEE, including C─C (284.8 eV) and C═O (286.6 eV), ascribed to the decomposition of PDOL polymer. In contrast, both these organic species and inorganic Li_2_CO_3_ (289.9 eV) are shown in LiFSI/PDOL‐BFE, which indicates its inorganic‐rich SEI nature. Moreover, the C═O originates from the reduction of C─O in PDOL chains by Li metal. The C═O content in LiFSI/PDOL‐BFE is only 15%, much less than that in LiFSI/PDOL‐DEE (53%) and LiFSI/PDOL (62%), indicating the inorganic‐rich SEI in LiFSI/PDOL‐BFE is stable enough to suppress parasitic reactions between Li metal and polymer matrix. F1s XPS spectra exhibit both organic S─F (688 eV) and inorganic LiF (684.8 eV) species in all three electrolytes (Figure 3b), derived from the decomposition of LiFSI. Notably, LiFSI/PDOL‐BFE contains substantially more LiF than the other systems (Figure S10, Supporting Information). This trend is further supported by N 1s spectra (Figure 3c), where LiFSI/PDOL‐BFE uniquely forms inorganic Li_3_N (396.1 eV), while LiFSI/PDOL and LiFSI/PDOL‐DEE only exhibit organic N─S (400.1 eV).

Inorganic‐rich SEI with high mechanical strength can depress the continuous lithium dendrite growth and promote uniform Li deposition, revealed by scanning electron microscope (SEM) and inserted optical images. In LiFSI/PDOL (Figure 3d), Li deposited at 5 mAh cm^−2^ exhibits a mossy, porous morphology, leaving large areas of uncovered Cu foil. This result is in an excessive thickness (37.5 µm, Figure 3g), significantly greater than the theoretical value (25 µm) for 5 mAh cm^−2^ Li metal. While LiFSI/PDOL‐DEE (Figure 3e) shows denser Li chunks with improved Cu coverage, numerous small pores persist, yielding a thickness (33.2 µm, Figure 3h) still far above the theoretical value. In stark contrast, LiFSI/PDOL‐BFE (Figure 3f) produces compact Li deposits with near‐zero porosity, complete Cu coverage, and minimal thickness (25.8 µm, Figure 3i), approaching the theoretical thickness.

### 2.3 High‐Voltage Performance and Li Metal Compatibility

**Figure 4:**
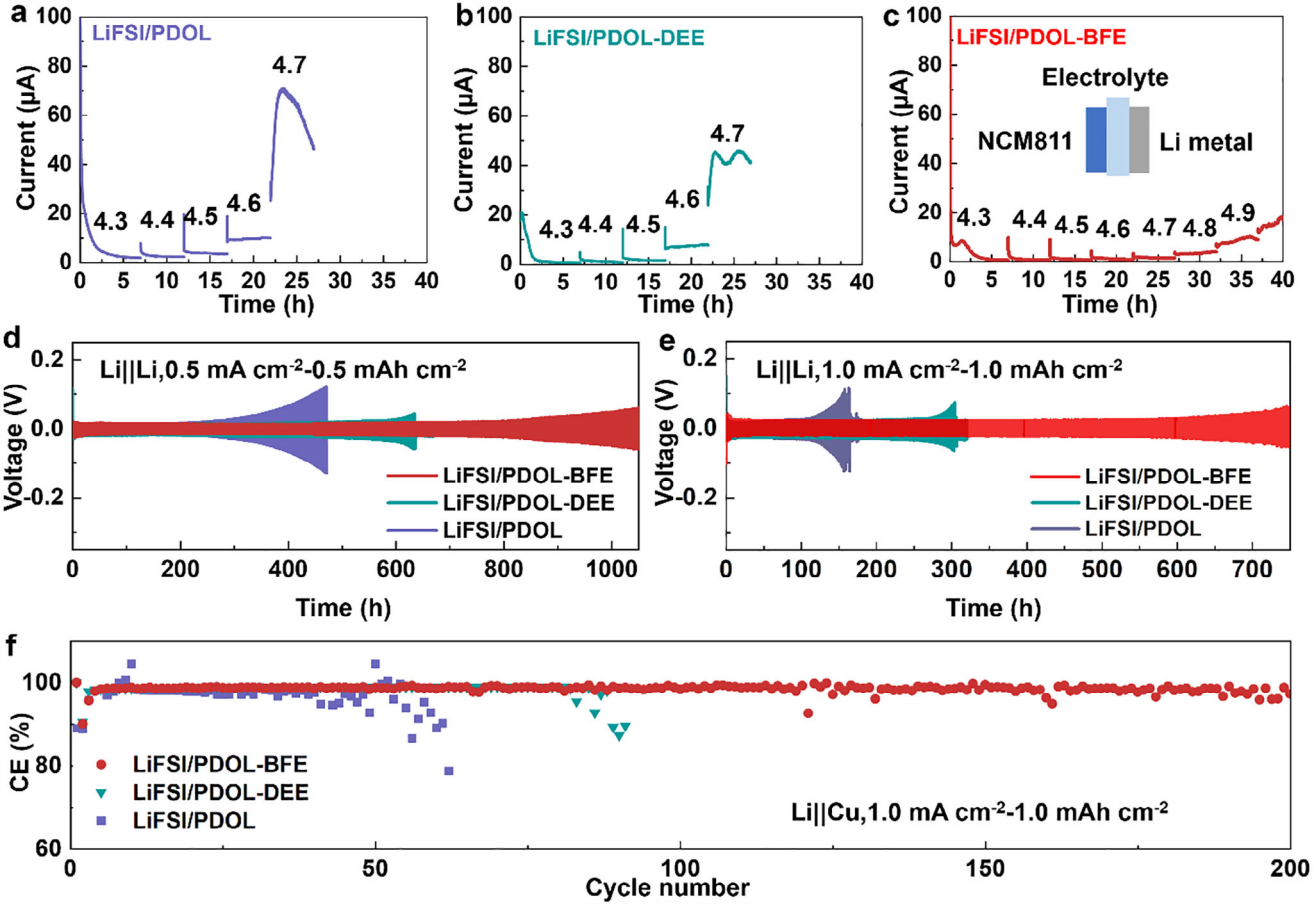
High‐voltage stability and Li metal efficiency of various electrolytes. a–c) Electrochemical floating tests with NCM_811_ cathodes. Galvanostatic plating/stripping profiles of Li||Li cells at 0.5 mA cm^−2^ under 0.5 mA h cm^−2^ capacity limitation d) and 1.0 mA cm^−2^ under 1.0 mA h cm^−2^ capacity limitation e). f) Coulombic efficiencies of Li||Cu cells at 1.0 mA cm^−2^ under 1.0 mA h cm^−2^ capacity limitation.

The inorganic‐rich CEI formed in LiFSI/PDOL‐BFE enables its desirable high‐voltage stability. An electrochemical floating test with Li||NCM_811_ cells was conducted to evaluate the electrolyte stability toward the high‐voltage cathode (**Figure** **4**a–c; Figure S11, Supporting Information). In LiFSI/PDOL, the leakage current after long‐time relaxation (7 h) is very low (<5 µA) when the applied potential is lower than 4.5 V. However, at higher potential (e.g., 4.7 V), the current rapidly increases to over 60 µA, ascribed to the electrolyte decomposition. The high‐voltage stability is slightly enhanced to 4.5 V in LiFSI/PDOL‐DEE but is still relatively poor. In contrast, the cell in LiFSI/PDOL‐BFE can stably operate at high voltage up to 4.7 V. Even at 4.9 V, the leakage current is less than 20 µA. Linear sweep voltammetry measurements on Li||Al cells further demonstrate the improved oxidation stability upon BFE incorporation, with upper voltages of 4.3 V in LiFSI/PDOL, 4.5 V in LiFSI/PDOL‐DEE, and 4.8 V in LiFSI/PDOL‐BFE (Figure S12, Supporting Information). Furthermore, LiFSI/PDOL‐BFE exhibits significantly enhanced ionic transport properties compared to LiFSI/PDOL‐DEE and LiFSI/PDOL (Figures S13 and S14, Supporting Information), demonstrating lower activation energies (0.27, 0.57, and 0.67 eV, respectively) and improved Li^+^ transference number (0.47, 0.34 and 0.29, respectively). These improvements can be attributed to the favorable solvation capability of BFE, which facilitates more efficient Li^+^ transport.

Inorganic‐rich SEI improves Li metal stability in LiFSI/PDOL‐BFE, demonstrated by Li||Cu and Li||Li cells with current density of 1 mA cm^−2^ and capacity limitation of 1 mAh cm^−2^. All Li||Cu cells experience a SEI formation process in the initial few cycles (CE<95%), but the CE will increase to a steady value after that (CE>97%, Figure 4d). In LiFSI/PDOL and LiFSI/PDOL‐DEE, the Li||Cu cells can only normally operate for less than 100 cycles. By comparison, the Li metal in LiFSI/PDOL‐BFE shows a much longer cycle life with a high average CE of 98.5% during 200 cycles, superior to reported PSSEs.^[^ ^7^ ^]^ Li||Li cells with other cycle conditions (e.g., 0.5 mA cm^−2^‐0.5 mAh cm^−2^, Figure S15, Supporting Information) also support the enhanced Li metal stability in LiFSI/PDOL‐BFE. Li||Li cells in both LiFSI/PDOL and LiFSI/PDOL‐DEE showcase a three‐step evolution process (Figure 4e). The initial uniform Li plating leads to a constant overpotential of ≈20 mV, followed by sharply increased overpotential due to the start formation of Li dendrites. Finally, the dendrite will short the battery, along with an abnormal overpotential decrease. The cells can only cycle for 120 and 280 h in LiFSI/PDOL and LiFSI/PDOL‐DEE, respectively. In contrast, LiFSI/PDOL‐BFE prolongs the cell life to more than 700 h without any short signal. Moreover, the Li||Li cell with LiFSI/PDOL‐BFE always shows less overpotential than LiFSI/PDOL‐DEE and LiFSI/PDOL at a wide range of current densities ranging from 0.5 to 5.0 mA cm^−2^ (Figure S16, Supporting Information).

### 2.4 Battery Performance

**Figure 5:**
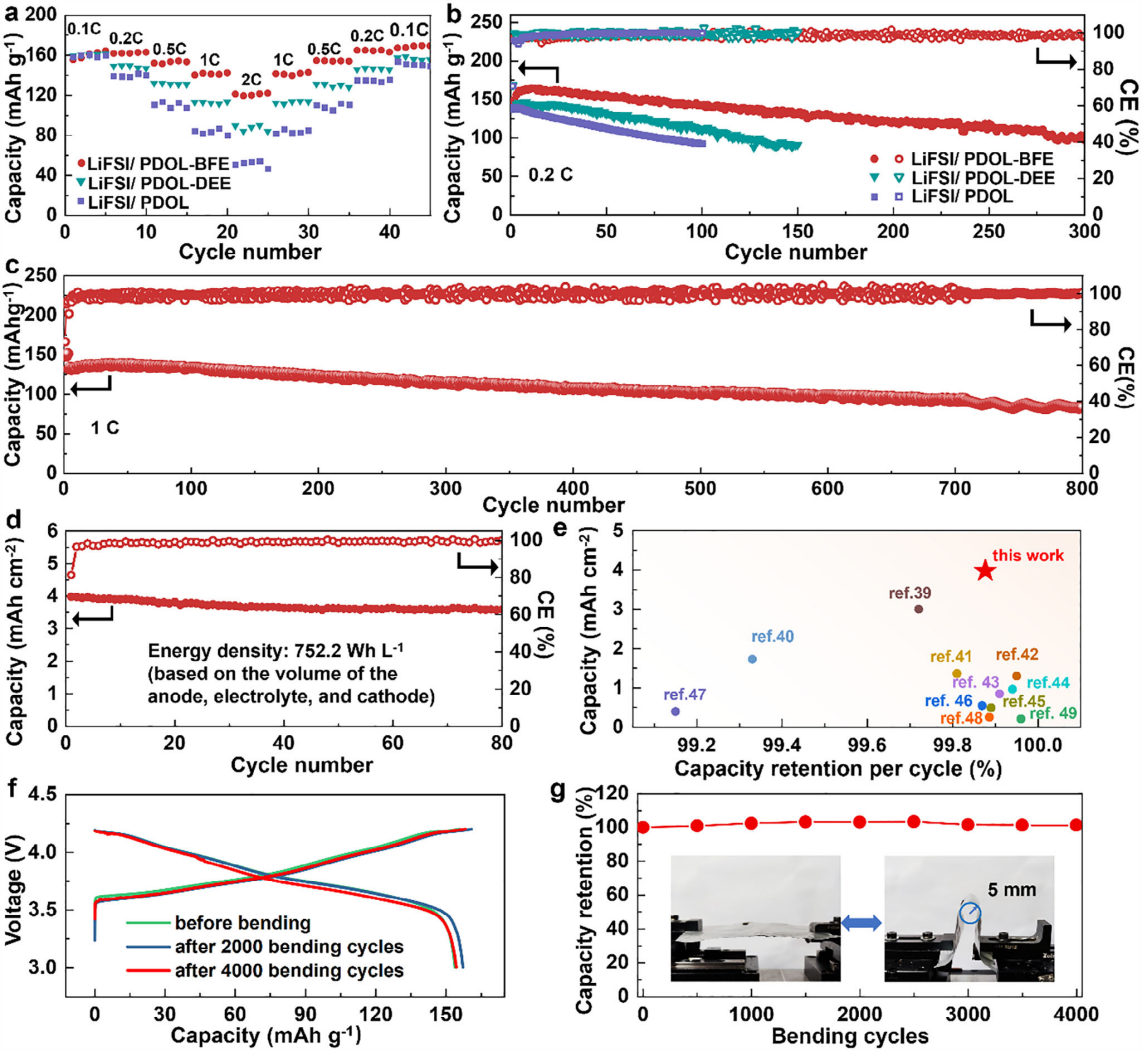
Performance of LMBs. The specific capacity at various C rates a), cycling durability at 0.2C b), and at 1C c) of low‐loading NCM_811_ (4 mg cm^−2^) in LiFSI/PDOL, LiFSI/PDOL‐DEE, and LiFSI/PDOL‐BFE. d) High‐loading (3.98 mAh cm^−2^) stability of the cathode in LiFSI/PDOL‐BFE and e) corresponding performance comparison in reported polymer electrolytes.^[^ ^39^, ^40^, ^41^, ^42^, ^43^, ^44^, ^45^, ^46^, ^47^, ^48^, ^49^ ^]^ Charge/discharge profiles f) and capacity retention g) in pouch cells during 4000 bending cycles (bending radius: 5 mm).

NCM_811_ with loading of ≈4 mg cm^−2^ is first used to prove the superiority of LiFSI/PDOL‐BFE. Electrochemical evaluation reveals significant differences in rate capability among the electrolytes. While LiFSI/PDOL delivers a respectable initial capacity of 160.0 mAh g^−1^ at 0.1C (1C = 150 mA g^−1^), its capacity dramatically declines to 54.4 mAh g^−1^ at 2C (**Figure** **5**a). LiFSI/PDOL‐DEE demonstrates improved high‐rate performance (89.4 mAh g^−1^ at 2C), though this remains suboptimal. In striking contrast, LiFSI/PDOL‐BFE achieves superior rate capability (121.9 mAh g^−1^ at 2C), representing a 2.2‐times enhancement over LiFSI/PDOL. This remarkable improvement is directly attributable to the electrolyte's superior Li⁺ conductivity and enhanced transference number. Low C‐rate cycling (e.g., 0.2C, Figure 5b), which is more challenging for electrolytes operating at high voltage, is chosen to further assess the long‐term stability of prepared electrolytes. The battery in all electrolytes experiences a capacity activation process in the initial few cycles, where the capacity increases slightly. After that, their capacity decays in a linear mode because of the continuous interphase growth. Notably, batteries in LiFSI/PDOL‐BFE with stable inorganic‐rich CEI/SEI interfaces demonstrate superior electrochemical performance, achieving both the highest average CE (99.34%) and the longest cycle life (234 cycles to 80% capacity retention). This represents a significant improvement over LiFSI/PDOL (98.60%, 56 cycles) and LiFSI/PDOL‐DEE (99.06%, 92 cycles), highlighting the critical role of interface stability in cycling performance. The LiFSI/PDOL‐BFE also endows the battery with desirable cycling stability at a higher C‐rate (e.g., 1C, Figure 5c), which remains 60% capacity after 800 cycles.

Commercial high‐loading NCM_811_ (3.98 mAh cm^−2^) is applied to evaluate the practical application potential of LiFSI/PDOL‐BFE. The battery shows a high energy density of 752.2 Wh L^−1^ based on the volume of Li metal anode, LiFSI/PDOL‐BFE PSSE, and cathode (Table S1, Supporting Information). Importantly, such a high‐loading battery can stably operate for over 80 cycles with a high capacity retention of 90.1% (Figure 5d; Figure S17, Supporting Information). The capacity retention per cycle is as high as 99.88%, which is the best among all reported PSSEs (Figure 5e). The pouch cell was finally assembled to assess the flexible application potential of LiFSI/PDOL‐BFE. The cell exhibits overlapped charge‐discharge curves during 4000 bending cycles under a low bending radius (5 mm, Figure 5f,g), demonstrating the high potential of LiFSI/PDOL‐BFE for flexible batteries.

## 3 Conclusion

In summary, we design a high‐voltage, Li‐metal‐compatible, and high‐conductive LiFSI/PDOL‐BFE PSSE through precise solvation structure engineering. The formation of unique atypical hydrogen bonds between BFE and PDOL promotes increased FSI^−^ anion participation in the Li^+^ solvation shell, thereby facilitating the formation of inorganic‐rich CEI and SEI layers. Consequently, the LiFSI/PDOL‐BFE shows ultrahigh oxidation stability of >4.7 V along with a high Li metal CE of 98.5%, superior to reported PSSEs. Solid‐state LMBs coupling with NCM_811_ cathodes retain 80% capacity after 480 cycles under high rate cycling at 1 C. Practical full cells with a high volumetric energy density of 752.2 Wh L^−1^ also exhibit record‐high capacity retention per cycle (99.88%). The LiFSI/PDOL‐BFE furthermore endows highly flexible pouch cells, showing almost identic capacity utilization after 4000 bending cycles. Due to the limited high‐voltage stability of the NCM_811_ cathode material employed in this study, our full‐cell evaluations were only conducted at 4.2 V. However, the promising electrochemical performance observed in these preliminary studies strongly suggests that the LiFSI/PDOL‐BFE will be particularly suitable for higher‐voltage cathode materials, such as Li/Mn‐rich layered oxides or LiNi_0.5_Mn_1.5_O_4_ spinel, which will be the focus of future investigations.

## Conflict of Interest

The authors declare no conflict of interest.

## Supporting information



Supporting Information

## Data Availability

The data that support the findings of this study are available in the supplementary material of this article.

## References

[1] C. V. Amanchukwu , Z. Yu , X. Kong , J. Qin , Y. Cui , Z. Bao , J. Am. Chem. Soc. 2020, 142, 7393. 32233433 10.1021/jacs.9b11056

[2] J. Chang , J. Shang , Y. Sun , L. K. Ono , D. Wang , Z. Ma , Q. Huang , D. Chen , G. Liu , Y. Cui , Y. Qi , Z. Zheng , Nat. Commun. 2018, 9, 4480. 30367063 10.1038/s41467-018-06879-7 PMC6203774

[3] T. Li , X.‐Q. Zhang , P. Shi , Q. Zhang , Joule 2019, 3, 2647.

[4] Z. Yu , H. Wang , X. Kong , W. Huang , Y. Tsao , D. G. Mackanic , K. Wang , X. Wang , W. Huang , S. Choudhury , Y. Zheng , C. V. Amanchukwu , S. T. Hung , Y. Ma , E. G. Lomeli , J. Qin , Y. Cui , Z. Bao , Nat. Energy 2020, 5, 526.

[5] Q.‐K. Zhang , X.‐Q. Zhang , J. Wan , N. Yao , T.‐L. Song , J. Xie , L.‐P. Hou , M.‐Y. Zhou , X. Chen , B.‐Q. Li , R. Wen , H.‐J. Peng , Q. Zhang , J.‐Q. Huang , Nat. Energy 2023, 8, 725.

[6] L. Chen , W. Li , L. Z. Fan , C. W. Nan , Q. Zhang , Adv. Funct. Mater. 2019, 29, 1901047.

[7] Y. H. Chen , P. Lennartz , K. L. Liu , Y. C. Hsieh , F. Scharf , R. Guerdelli , A. Buchheit , M. Grünebaum , F. Kempe , M. Winter , G. Brunklaus , Adv. Funct. Mater. 2023, 33, 2300501.

[8] T. Deng , L. Cao , X. He , A.‐M. Li , D. Li , J. Xu , S. Liu , P. Bai , T. Jin , L. Ma , M. A. Schroeder , X. Fan , C. Wang , Chem 2021, 7, 3052.

[9] X. He , Y. Ni , Y. Hou , Y. Lu , S. Jin , H. Li , Z. Yan , K. Zhang , J. Chen , Angew. Chem., Int. Ed. 2021, 60, 22672. 10.1002/anie.202107648 34423516

[10] M. Jia , P. Wen , Z. Wang , Y. Zhao , Y. Liu , J. Lin , M. Chen , X. Lin , Adv. Funct. Mater. 2021, 31, 2101736.

[11] Q. Liu , G. Yang , X. Li , S. Zhang , R. Chen , X. Wang , Y. Gao , Z. Wang , L. Chen , Energy Storage Mater. 2022, 51, 443.

[12] J. Wang , K. Wang , Y. Xu , ACS Nano 2021, 15, 19026. 34842431 10.1021/acsnano.1c09194

[13] Y. G. Cho , C. Hwang , D. S. Cheong , Y. S. Kim , H. K. Song , Adv. Mater. 2019, 31, 1804909. 10.1002/adma.201804909 30387233

[14] F. He , W. Tang , X. Zhang , L. Deng , J. Luo , Adv. Mater. 2021, 33, 2105329. 10.1002/adma.202105329 34536045

[15] K. He , S. H. Cheng , J. Hu , Y. Zhang , H. Yang , Y. Liu , W. Liao , D. Chen , C. Liao , X. Cheng , Z. Lu , J. He , J. Tang , R. K. Y. Li , C. Liu , Angew. Chem., Int. Ed. 2021, 60, 12116. 10.1002/anie.202103403 33723915

[16] C. Niu , W. Luo , C. Dai , C. Yu , Y. Xu , Angew. Chem., Int. Ed. 2021, 60, 24915. 10.1002/anie.202107444 34296502

[17] Y.‐F. Huang , T. Gu , G. Rui , P. Shi , W. Fu , L. Chen , X. Liu , J. Zeng , B. Kang , Z. Yan , F. J. Stadler , L. Zhu , F. Kang , Y.‐B. He , Energy Environ. Sci. 2021, 14, 6021.

[18] X. Lin , J. Yu , M. B. Effat , G. Zhou , M. J. Robson , S. C. T. Kwok , H. Li , S. Zhan , Y. Shang , F. Ciucci , Adv. Funct. Mater. 2021, 31, 2010261.

[19] M. Ma , F. Shao , P. Wen , K. Chen , J. Li , Y. Zhou , Y. Liu , M. Jia , M. Chen , X. Lin , ACS Energy Lett. 2021, 6, 4255.

[20] C. Z. Zhao , Q. Zhao , X. Liu , J. Zheng , S. Stalin , Q. Zhang , L. A. Archer , Adv. Mater. 2020, 32, 1905629. 10.1002/adma.201905629 32053238

[21] Q. Zhao , X. Liu , S. Stalin , K. Khan , L. A. Archer , Nat. Energy 2019, 4, 365.

[22] Q. Ma , J. Yue , M. Fan , S. J. Tan , J. Zhang , W. P. Wang , Y. Liu , Y. F. Tian , Q. Xu , Y. X. Yin , Y. You , A. Luo , S. Xin , X. W. Wu , Y. G. Guo , Angew. Chem., Int. Ed. 2021, 60, 16554. 10.1002/anie.202103850 33955135

[23] Y. Wang , T. Li , X. Yang , Q. Yin , S. Wang , H. Zhang , X. Li , Adv. Energy Mater. 2023, 14, 2303189.

[24] H. Cheng , J. Zhu , H. Jin , C. Gao , H. Liu , N. Cai , Y. Liu , P. Zhang , M. Wang , Mater. Today Energy 2021, 20, 100623.

[25] H. Yang , B. Zhang , M. Jing , X. Shen , L. Wang , H. Xu , X. Yan , X. He , Adv. Energy Mater. 2022, 12, 2201762.

[26] S. Liu , B. Wu , S. Huang , Z. Lin , H. Song , L. Du , Z. Liang , Z. Cui , Adv. Energy Mater. 2024, 15, 2402848.

[27] C. Xie , M. Rong , Q. Guo , Z. Wei , Z. Chen , Q. Huang , Z. Zheng , Adv. Mater. 2024, 36, 2406368. 10.1002/adma.202406368 38896050

[28] C. Zhang , Z. Niu , J. Bae , L. Zhang , Y. Zhao , G. Yu , Energy Environ. Sci. 2021, 14, 931.

[29] H. Yang , M. Jing , L. Wang , H. Xu , X. Yan , X. He , Nano‐Micro Lett. 2024, 16, 127. 10.1007/s40820-024-01354-z PMC10881957 38381226

[30] X. Yang , M. Jiang , X. Gao , D. Bao , Q. Sun , N. Holmes , H. Duan , S. Mukherjee , K. Adair , C. Zhao , J. Liang , W. Li , J. Li , Y. Liu , H. Huang , L. Zhang , S. Lu , Q. Lu , R. Li , C. V. Singh , X. Sun , Energy Environ. Sci. 2020, 13, 1318.

[31] Z. Shen , J. Zhong , J. Chen , W. Xie , K. Yang , Y. Lin , J. Chen , Z. Shi , Chin. Chem. Lett. 2023, 34, 107370.

[32] S. Zheng , Y. Chen , K. Chen , S. Yang , R. Bagherzadeh , Y.‐E. Miao , T. Liu , J. Mater. Chem. A 2022, 10, 19641.

[33] Z. Geng , Y. Huang , G. Sun , R. Chen , W. Cao , J. Zheng , H. Li , Nano Energy 2022, 91, 106679.

[34] X. Liu , D. Wang , Z. Zhang , G. Li , J. Wang , G. Yang , H. Lin , J. Lin , X. Ou , W. Zheng , Small 2024, 20, 2404879. 10.1002/smll.202404879 39101287

[35] Y. Wu , J. Ma , H. Jiang , L. Wang , F. Zhang , X. Feng , H. Xiang , Mater. Today Energy 2023, 32, 10123934.

[36] Y. Bai , W. Ma , W. Dong , Y. Wu , X. Wang , F. Huang , ACS Appl. Mater. Interfaces 2023, 15, 26834. 37222274 10.1021/acsami.3c04234

[37] J. P. Hoffknecht , A. Wettstein , J. Atik , C. Krause , J. Thienenkamp , G. Brunklaus , M. Winter , D. Diddens , A. Heuer , E. Pailard , Adv. Energy Mater. 2023, 13, 2202789.

[38] L. Wang , Y. L. Liu , M. S. Wang , Langmuir 2023, 39, 357. 36524998 10.1021/acs.langmuir.2c02594

[39] P. Xu , Y. C. Gao , Y. X. Huang , Z. Y. Shuang , W. J. Kong , X. Y. Huang , W. Z. Huang , N. Yao , X. Chen , H. Yuan , C. Z. Zhao , J. Q. Huang , Q. Zhang , Adv. Mater. 2024, 36, 2409489. 10.1002/adma.202409489 39210646

[40] G. Ye , L. Zhu , Y. Ma , M. He , C. Zheng , K. Shen , X. Hong , Z. Xiao , Y. Jia , P. Gao , Q. Pang , J. Am. Chem. Soc. 2024, 146, 27668. 39323328 10.1021/jacs.4c09062

[41] P. Wang , Y. Liu , J. Cui , L. Zhao , D. Li , Y. Du , H. Li , Adv. Funct. Mater. 2024, 35, 2414430.

[42] S. Chai , Y. Zhong , Y. Wang , Q. He , A. Azizi , L. Chen , X. Ren , W. Wei , S. Liang , Z. Chang , A. Pan , Adv. Energy Mater. 2023, 14, 2303020.

[43] W. Zhang , V. Koverga , S. Liu , J. Zhou , J. Wang , P. Bai , S. Tan , N. K. Dandu , Z. Wang , F. Chen , J. Xia , H. Wan , X. Zhang , H. Yang , B. L. Lucht , A.‐M. Li , X.‐Q. Yang , E. Hu , S. R. Raghavan , A. T. Ngo , C. Wang , Nat. Energy 2024, 9, 386.

[44] C. Wang , H. Liu , Y. Liang , D. Li , X. Zhao , J. Chen , W. Huang , L. Gao , L. Z. Fan , Adv. Funct. Mater. 2022, 33, 2209828.

[45] S. J. Yang , H. Yuan , N. Yao , J. K. Hu , X. L. Wang , R. Wen , J. Liu , J. Q. Huang , Adv. Mater. 2024, 36, 2405086. 10.1002/adma.202405086 38940367

[46] X. Wang , L. Xu , M. Li , Y. Hu , N. Wang , Y. Meng , K. Yang , K. Deng , Energy Storage Mater. 2024, 73, 103778.

[47] Y. Zheng , C. Wang , R. Zhang , S. Dai , H. Xie , J. Cui , X. Fang , Energy Storage Mater. 2023, 57, 540.

[48] R. Fang , B. Xu , N. S. Grundish , Y. Xia , Y. Li , C. Lu , Y. Liu , N. Wu , J. B. Goodenough , Angew. Chem., Int. Ed. 2021, 60, 17701. 10.1002/anie.202106039 34192402

[49] S. Chai , Y. Zhang , Y. Wang , Q. He , S. Zhou , A. Pan , eScience 2022, 2, 494.

