# Supplementary material for: Atypical Hydrogen Bond Interaction Enables Anion‐Rich Solvation Structure in Polymer Electrolytes for High‐Voltage Flexible Lithium Metal Batteries
Source: Adv Sci (Weinh). 2025 Jun 11;12(34):e07007. doi: 10.1002/advs.202507007 (PMC12442591; doi:10.1002/advs.202507007)
Supplement: Supplementary file 1 — Supporting Information [file ADVS-12-e07007-s001.docx]

**Supplementary Information**

**Atypical Hydrogen Bond Interaction Enables Anion-Rich Solvation Structure in Polymer Electrolytes for High-Voltage Flexible Lithium Metal Batteries**

*Shujing Wen^+^, Junhua Zhou^+^, Guangzhao Zhang^+^, Qingrong Wang, Chao Luo, Ruo Wang, Pengxian Li, Chaoyang Wang, Xiaoxiong Xu, Yonghong Deng*, Jian Chang*, Zijian Zheng**

Ms. S. Wen, Dr. J. Zhou, Prof. Z. J. Zheng

Department of Applied Biology and Chemical Technology, Faculty of Science, The Hong Kong Polytechnic University, Hung Hom, Hong Kong SAR, China

Ms. S. Wen, Dr. G. Zhang, Dr. Q. Wang, Dr. C. Luo, Dr. R. Wang, Prof. X. Xu, Prof. Y. H. Deng, Prof. J. Chang

Department of Materials Science and Engineering, School of Innovation and Entrepreneurship, Key University Laboratory of Highly Efficient Utilization of Solar Energy and Sustainable Development of Guangdong, Southern University of Science and Technology, Shenzhen 518055, China

Mr. P. Li, Prof. J. Chang

Dongguan Key Laboratory of Interdisciplinary Science for Advanced Materials and Large-Scale Scientific Facilities, School of Physical Sciences, Great Bay University, Dongguan, Guangdong, 523000, China

Prof. C. Wang

Research Institute of Materials Science, South China University of Technology, Guangzhou 510640, China

Prof. Z. J. Zheng

Research Institute for Smart Energy, The Hong Kong Polytechnic University, Hung Hom, Hong Kong SAR, China

Research Institute for Intelligent Wearable Systems, The Hong Kong Polytechnic University, Hung Hom, Hong Kong SAR, China

PolyU-Daya Bay Technology and Innovation Research Institute, Huizhou, Guangdong, 516083, China

PolyU-Wenzhou Technology and Innovation Research Institute, Wenzhou, Zhejiang, China

*Corresponding Authors: tczzheng@polyu.edu.hk (Z. Zheng), changj@gbu.edu.cn (J. Chang), and yhdeng08@163.com (Y. Deng).

**Experimental Procedures**

**Experimental Materials**

2-fluoro-ethanol, N-methyl pyrrolidone (NMP), 1-bromo-2-fluoro-ethane, ethyl ether, diethyl ether (DEE), potassium hydroxide (KOH), anhydrous magnesium sulfate (MgSO_4)_, calcium hydride (CaH_2_), and lithium hydride (LiH) were purchased from Aladdin (Shanghai, China). 1,3-dioxolane (DOL) and lithium bis(fluorosulfonyl)imide (LiFSI), monocrystal LiNi_0.8_Co_0.1_Mn_0.1_O_2_ (NCM_811_) power, and commercial NCM_811_ cathode were purchased from Capchem (Shenzhen, China). Carbon black (super P, TIMCAL) and polyvinylidene difluoride (PVDF) were received from Kejing (Shenzhen, China).

**Preparation of bis(2-fluoroethyl) ether (BFE)**

64 g 2-fluoro-ethanol (1 mol) was dissolved in 60 mL NMP. The above solution was slowly added into a round-bottom flask which contained 70.8 g KOH (1.2 mol) at -20 ℃. Then, 127 g 1-bromo-2-fluoro-ethane (1 mol) was added while stirring. The mixture was stirred at -20 ℃ for 12 h and at room temperature for another 24h. After the reaction, a suction filter was used to separate the precipitated white solid and the residual solution. Ethyl ether was added to the residual solution to extract the product. Then, brine was used to wash the solution three times. The ethyl ether was removed via rotary evaporation. The water of the solution was removed by anhydrous MgSO_4_, CaH_2_, and LiH. Finally, the product was collected by atmospheric distillation at 135 ℃ and then stored in a glove box containing a 4 Å molecular sieve.

**Preparation of electrolyte**

Various concentrations of LiFSI and BFE were completely dissolved into DOL. It should be noted that the polymer electrolyte with an optimal ratio of 50 vol% BFE, 2.0 M LiFSI was used for all electrochemical testing and battery fabrication. The LiFSI/PDOL-DEE was prepared by in situ polymerization of 50 vol% DEE and 2.0 M LiFSI in DOL. In addition, the PDOL electrolyte was also prepared by dissolving 2.0 M LiFSI into DOL solvents. All polymer electrolytes were prepared inside an argon-filled glovebox with stringently low concentrations of oxygen and moisture (< 1.0 ppm).

**Assembling of quasi-solid polymer electrolyte batteries**

The cathode was prepared by slurry-coating a uniformly dispersed mixture of NCM_811_, PVDF binders, and Super P additives (80:10:10) on carbon-coated aluminum foils and drying in a vacuum oven at 80 ℃ for 12 h. For high cate performance full cell, LATP-coated NCM_811_ cathode is used.

The coin cells with PSSE were fabricated by in-situ polymerization. The Celgard 2500 separator was used between the cathode and Li foil. The precursor solution was injected into the cell before polymerization, and the batteries were filled with precursor solution, indicating great interface compatibility between electrolyte and electrodes. Succeeding, the batteries were left to stand for 24 h.

**Characterization**

Raman spectroscopy was obtained using a Thermo Scientific™ DXR3 Raman Microscope with a 532 nm laser to analyze the solvated structure of the electrolytes. The chemical structures of electrolytes were analyzed by Fourier transform infrared spectroscopy (FTIR, Vector 33-MIR) in a glove box. The molecular structures of PDOL-BFE were deeply investigated by nuclear magnetic resonance analysis (NMR, AVANCE Ⅲ 400 M) using deuterated chloroform as the solvent to lock fields. The surface morphology of the Li metal anodes was examined by field-emission scanning electron microscopy (FE-SEM, Tescan MIRA3, Czech Republic). The interfacial composition of cathodes and anodes was studied by X-ray photoelectron spectroscopy (XPS, PHI 5000 Versaprobe Ⅲ) with monochromatic Al K𝛼 radiation (1486.6). These electrode samples for XPS analysis were rinsed with DOL solvents for 24 h and transferred into a vacuum chamber after drying out. Cryo-TEM experiments were performed on a Thermo Fisher Titan Krios G2 transmission electron microscope operated at 5 kV. Cryo-TEM images were acquired by a Gatan K3 direct-detection camera in the electron counting mode. The NCM_811_ cathode samples were dipped in DOL solvent for 24 h to remove the electrolytes completely.

**Electrochemical measurements**

The ion conductivity under different temperatures, Li^+^ transference number, electrochemical floating analysis, and electrochemical stability window were tested on a multi-channel potentiostatic electrochemical workstation (Solartron 1470E). Electrochemical impedance spectroscopy (EIS) was used to measure the ionic conductivity with a frequency range from 0.1Hz to 1M Hz and an AC perturbation voltage of 5 mV. Two stainless-steel blocking electrodes were used to assemble the coin cell. The ionic conductivity (𝜎) of the electrolyte was calculated by the following equation:

𝜎= L/(S ×R)

where L (cm) is the thickness of the electrolyte, R (Ω) is the bulk resistance of the electrolyte, and S (cm^2^) is the effective contacting area between the electrolyte and the SS electrode. The Li^+^ transference number (t_Li_ ^+^) of the electrolyte was measured through the chronoamperometry method with a Li||Li symmetric cell. The t_Li_^+^ can be calculated by the following equation:

$$t_{{Li}^{+}}=\frac{I_{ss}\left( \Delta V-I_{0}R_{0} \right)}{I_{0}\left( \Delta V-I_{ss}R_{ss} \right)}$$

Where ΔV is the DC polarization voltage (5 mV), I_0_ and I_ss_ are the initial and steady-state current before and after polarization, while R_0_ and R_ss_ are the interfacial resistances of the symmetric cell before and after polarization, respectively. Linear sweep voltammograms (LSV) were used to measure the electrochemical stability window of the electrolyte with an asymmetric cell of Al||Li in the voltage range of 1 V to 7 V with a scanning rate of 0.5 mV s^-1^. The electrochemical floating experiments were conducted in Li||NCM_811_ cells. The cells were charged to 4.2 V first, then held at higher voltages until 5.0 V, each for a period of 5 h. The Coulombic efficiency test was carried out on Li||Cu cell. The Li||Li symmetric cells were assembled to test out the long-cycle performances for electrolytes. The galvanostatic cycling measurement of all the Li metal batteries was performed on Neware battery testing systems (CT2001A). All cells are texted out at 30 ℃. For pouch cell assembly, carbon paper was chosen as the cathode current collector and Cu-PET fabric was chosen as the anode current collector. Using a roll-pressing technique, the flexible anode was manufactured by laminating a 50 μm-thick lithium metal foil onto Cu-PET fabric substrate. The pouch cell was encapsulated using an aluminum laminate composite foil.

**Theoretical calculation**

All the density functional theory (DFT) calculations were carried out by using Gaussian 09W program. The orbital levels and electrostatic potentials (ESPs) of the molecules were evaluated. Geometric configurations were optimized at the B3LYP-D3 level with a 6-311+ G (d, p) basis set. The binding energy between BFE and PDOL was calculated following geometry optimizations. Similarly, binding energies of Li⁺-PDOL and Li⁺-PDOL-BFE complexes were determined by optimizing the full complexes both with and without Li⁺, where the absence of Li⁺ represents complete ionic dissociation at infinite separation distance.


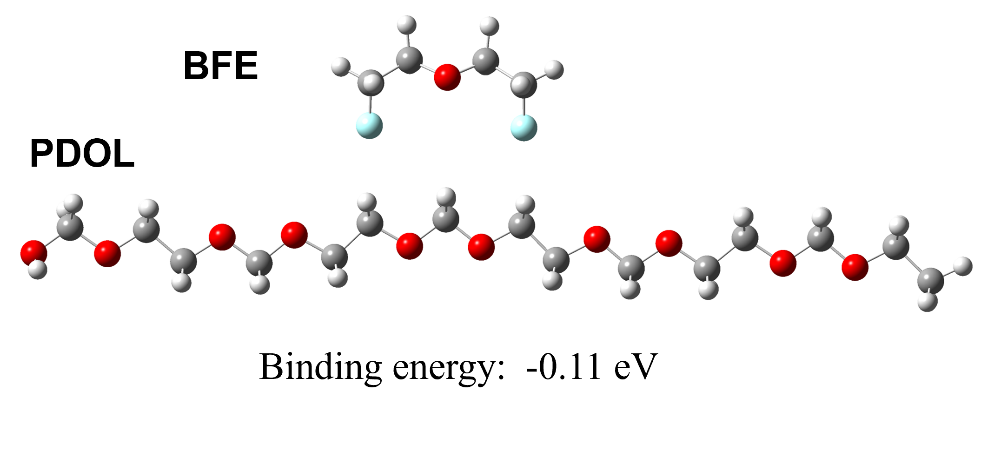


**Figure S1.** DFT calculated binding energy of BFE and PDOL chain.


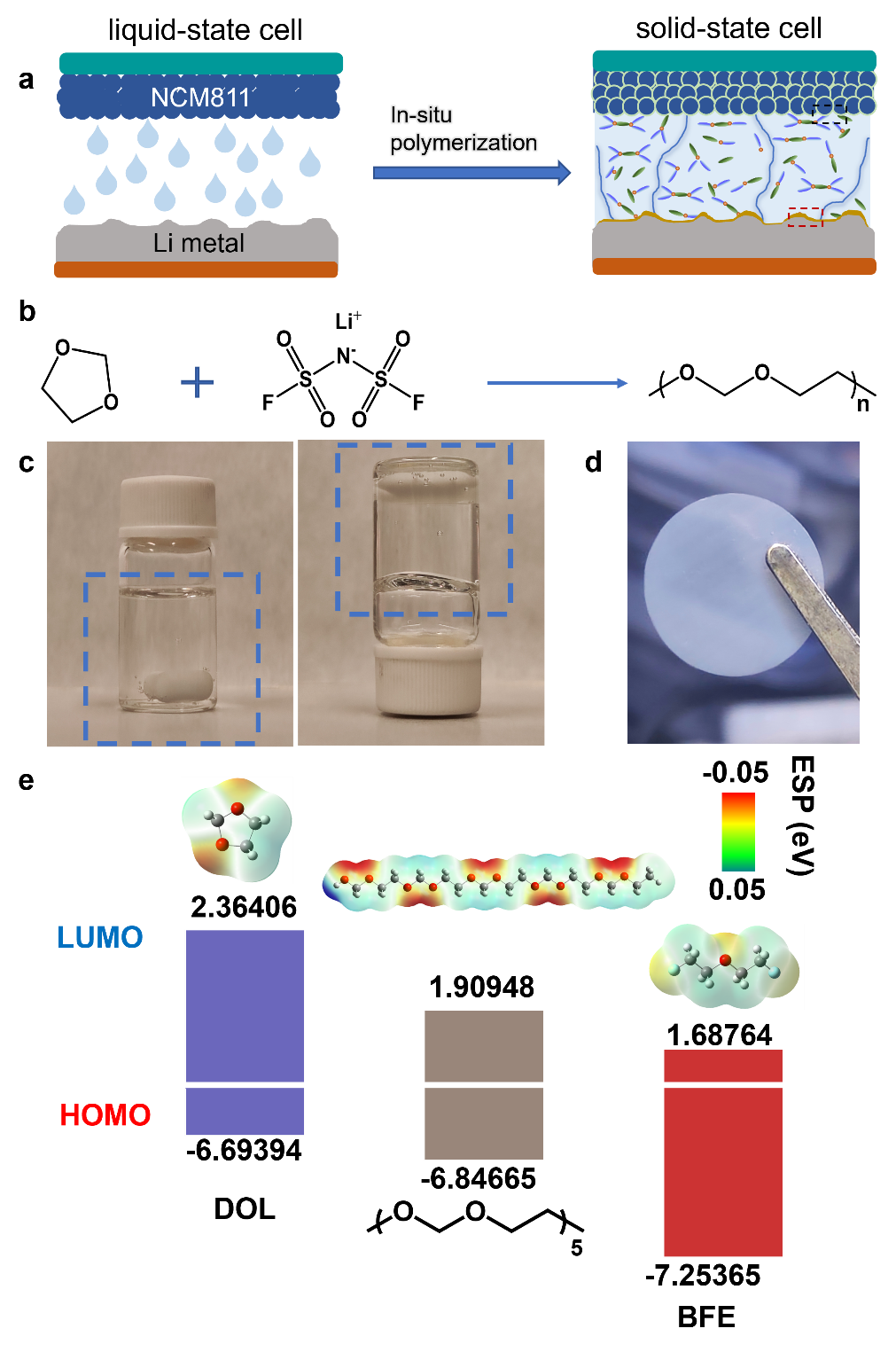


**Figure S2**. (a) Schematic of in-situ polymerization of LiFSI/PDOL-BFE electrolyte. (b) The reaction mechanism illustrates the polymerization of DOL. (c) Digital photos of the precursor solution before and after polymerization. (d) The electrolyte becomes a uniform and transparent gel after dissolving 2 M LiFSI and 50 vol% BFE in DOL. (e) Comparison of chemical structures and electrostatic potentials (ESP) of DOL, PDOL and BFE.


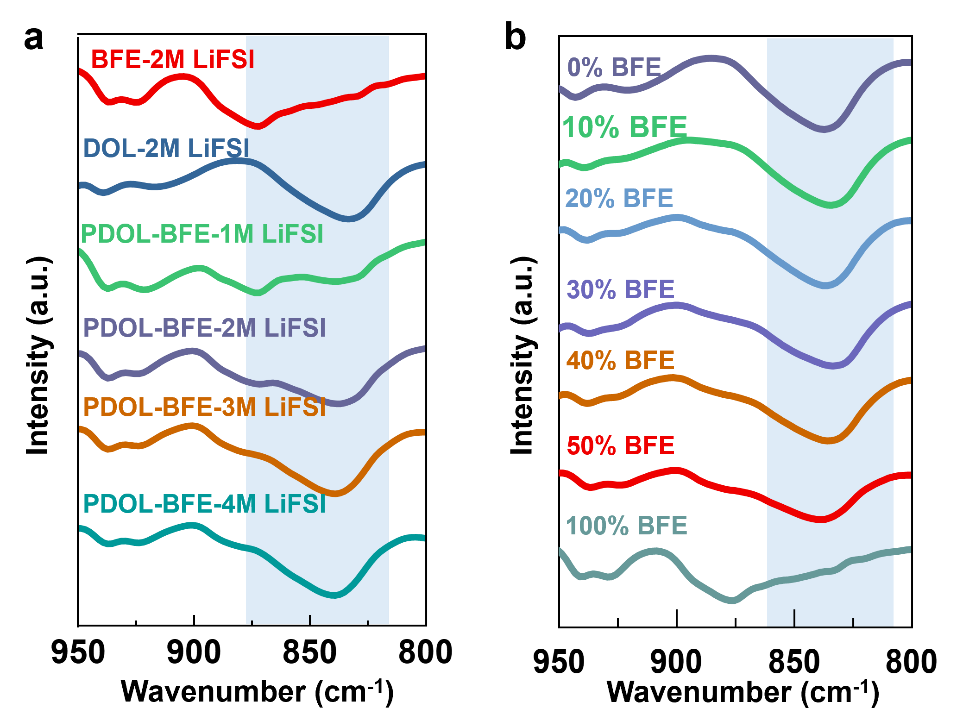


**Figure S3**. The FTIR spectra of electrolytes with various concentrations of LiFSI (a) and BFE (b).

**Note:** The influence of the concentration of LiFSI has been studied. The FTIR result suggests long-chain C-C vibration peaks at 835 cm^-1^ appear with 2 M LiFSI in gel electrolytes, while the DOL cannot be fully polymerized with 1 M LiFSI. And the addition of 50% BFE does not compromise the polymerization of DOL.


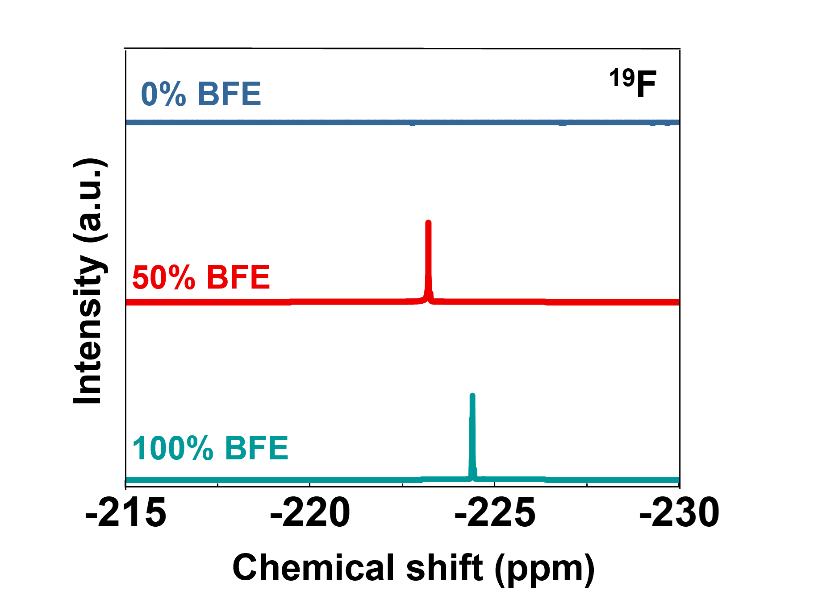


**Figure S4.** The ^19^F NMR spectra of 0% BFE, 50% BFE and 100% BFE.


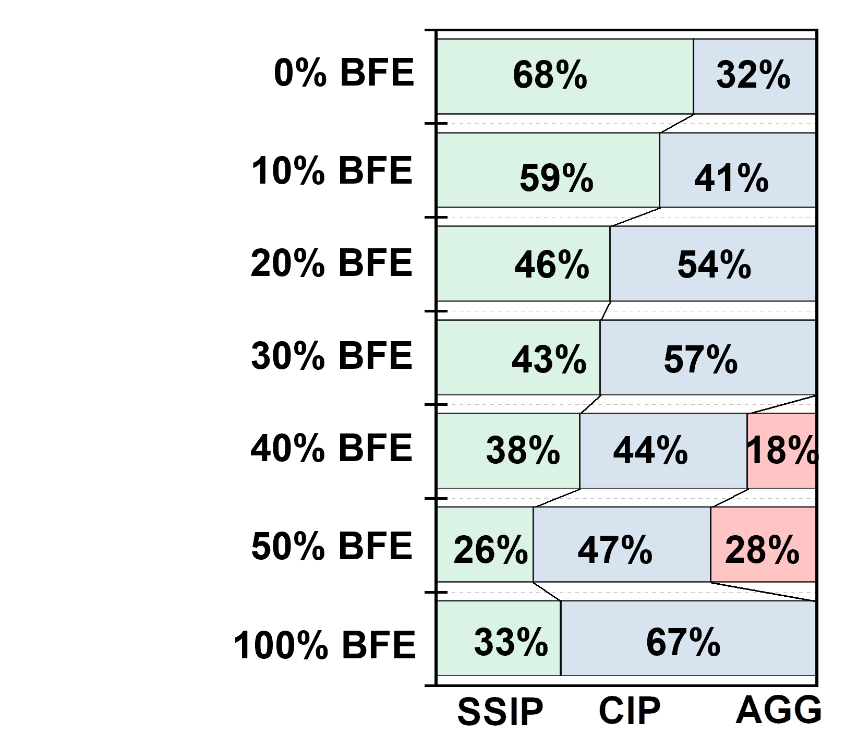


**Figure S5.** SSIP, CIP, and AGG ratio of various electrolytes based on Raman results.


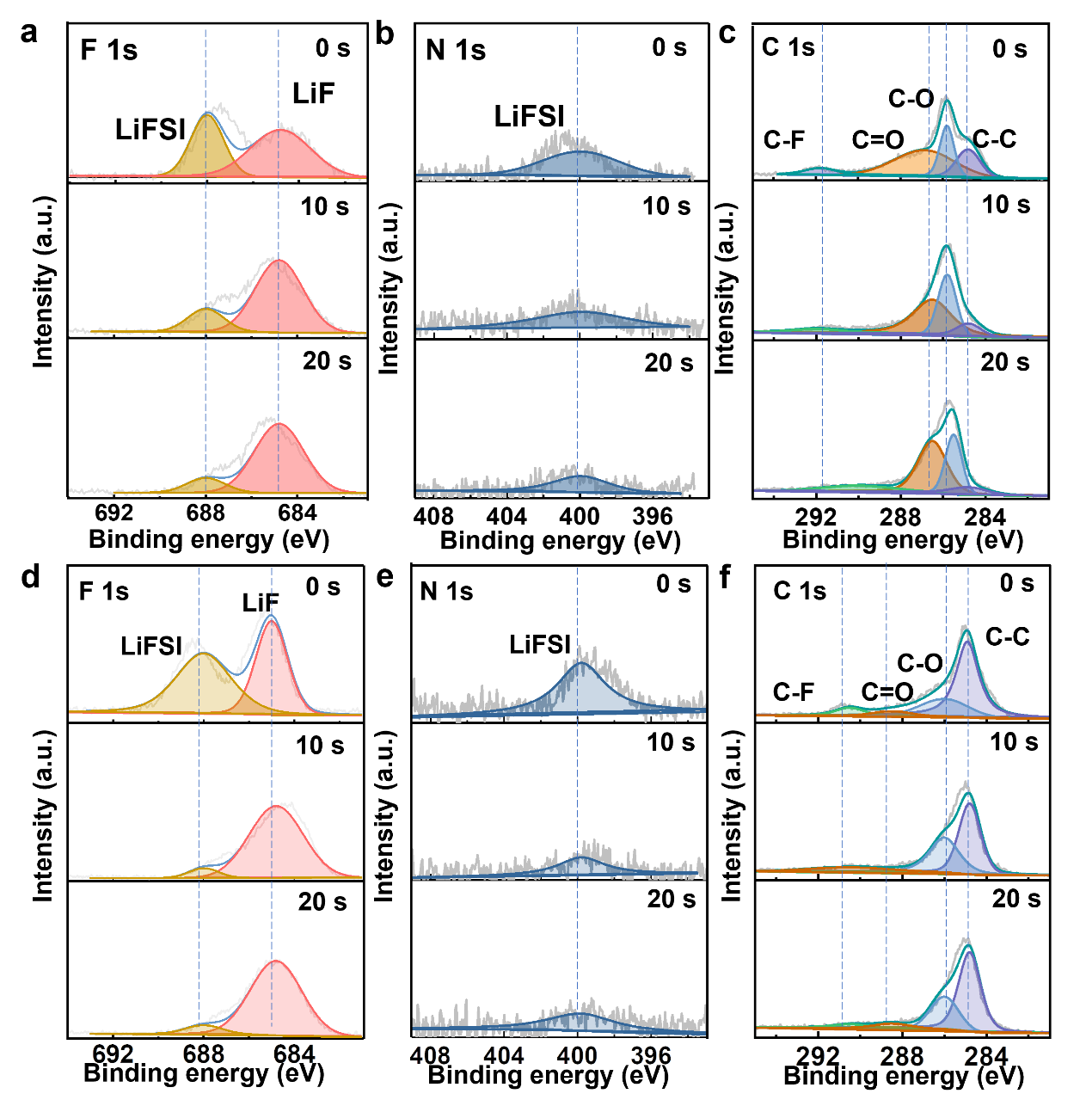


**Figure S6.** The XPS results of CEI in LiFSI/PDOL (a-c) and LiFSI/PDOL-DEE (d-f) with various etching times.


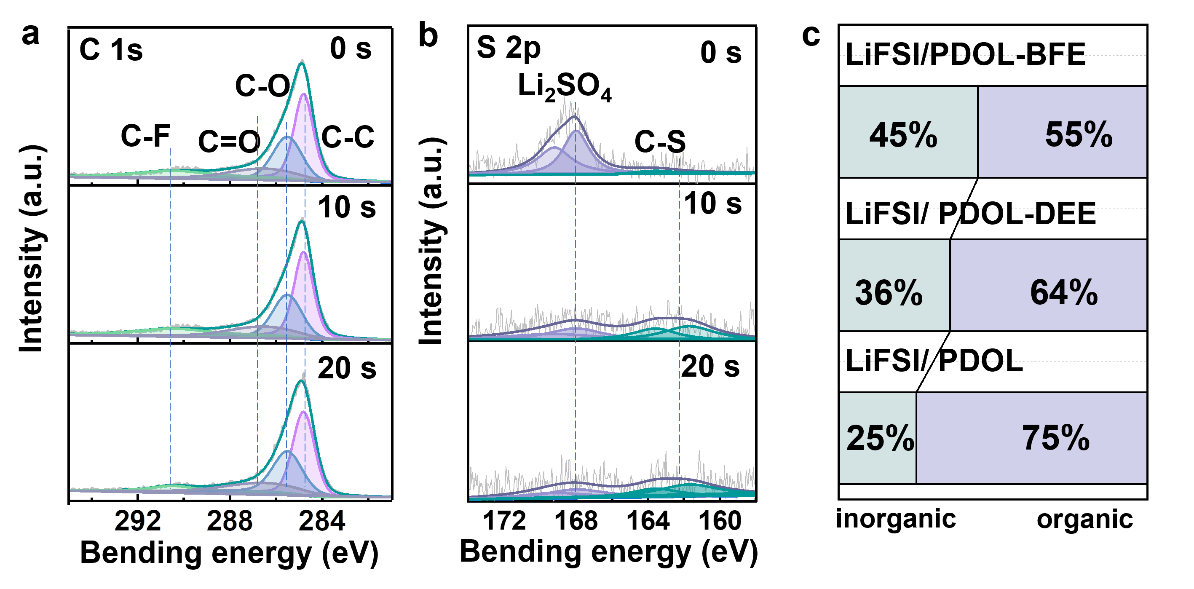


**Figure S7**. C 1s (a) and S 2p (b) XPS result of CEI component in LiFSI/PDOL-BFE. (c) Inorganic and organic component ratio of CEI based on XPS results.


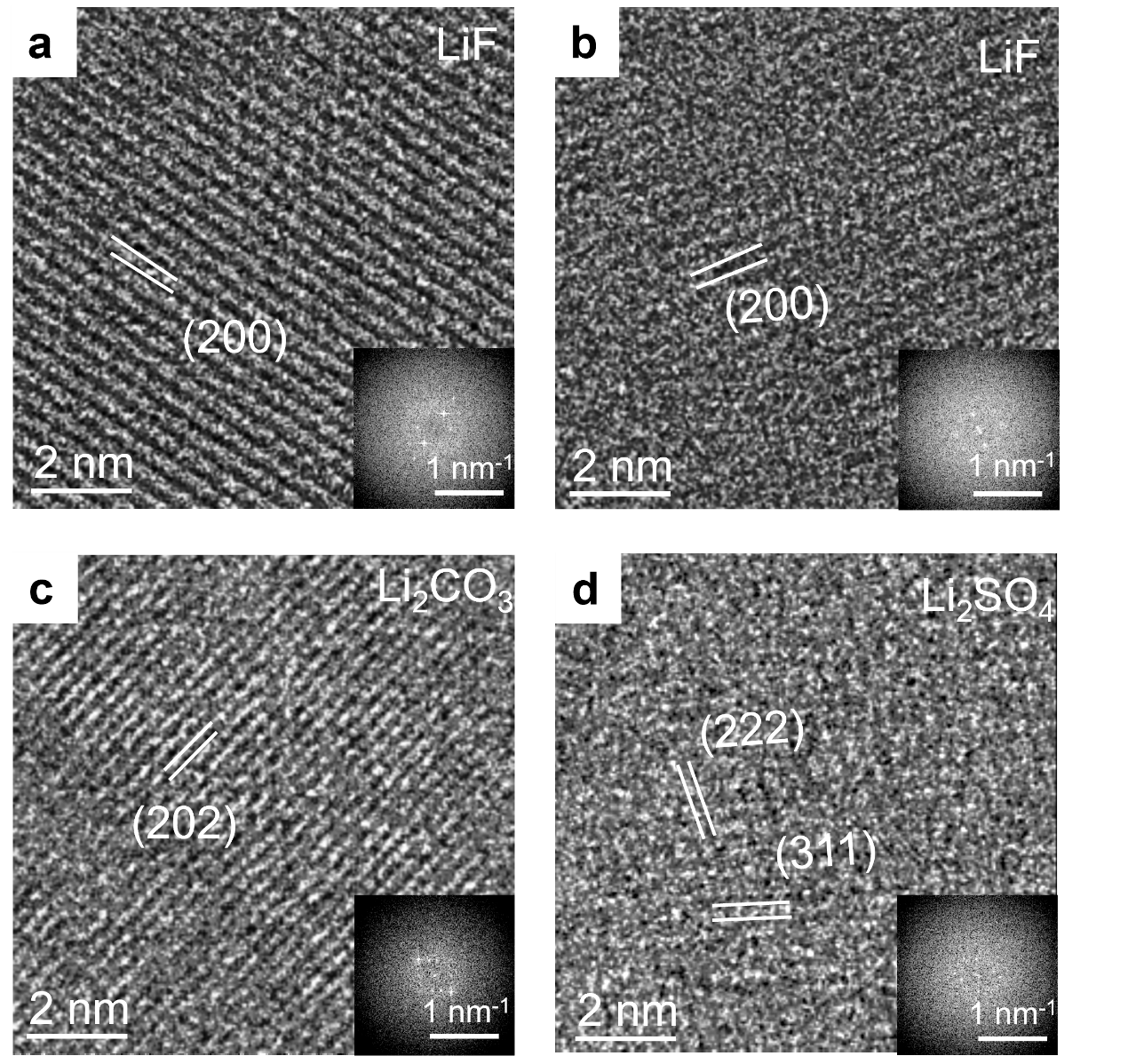


**Figure S8.** Cryo-TEM images and the corresponding FFT patterns of the CEI layer in LiFSI/PDOL electrolyte (a) and LiFSI/PDOL-DEE (b-d).


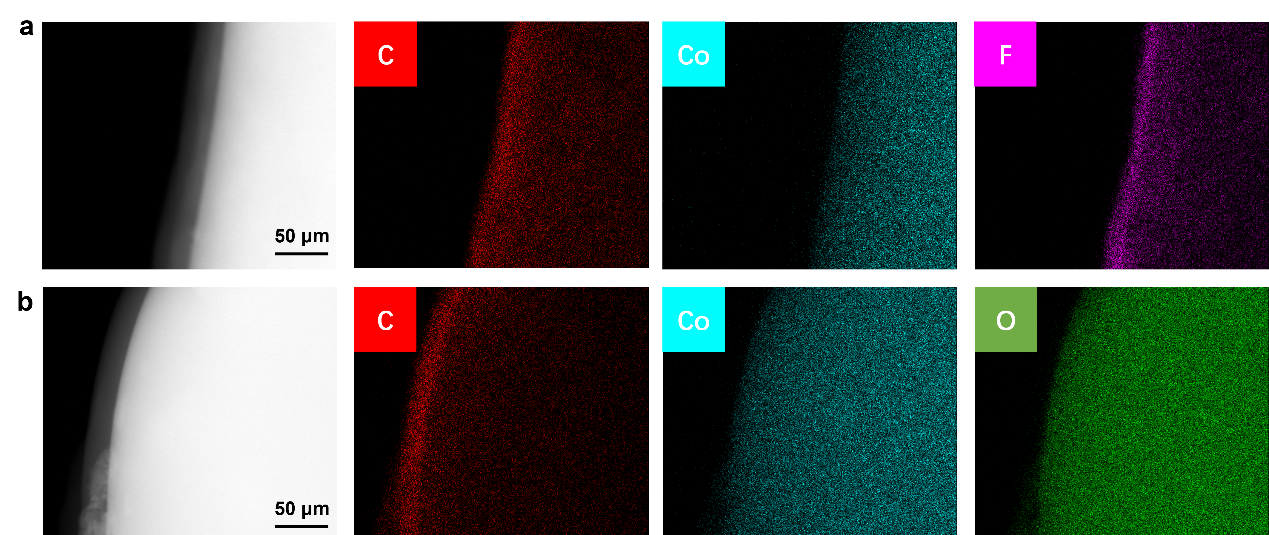


**Figure S9.** TEM images and EELS elemental mapping images of the NCM_811_ cathode with LiFSI/PDOL-BFE (a) and LiFSI/PDOL (b) electrolyte after 50 cycles.


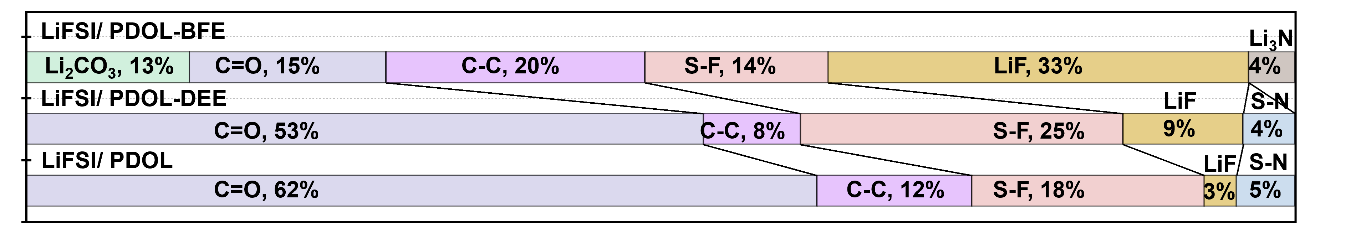


**Figure S10.** The proportion of each component in the SEI in LiFSI/PDOL-BFE, LiFSI/PDOL-DEE, and LiFSI/PDOL based on XPS results.


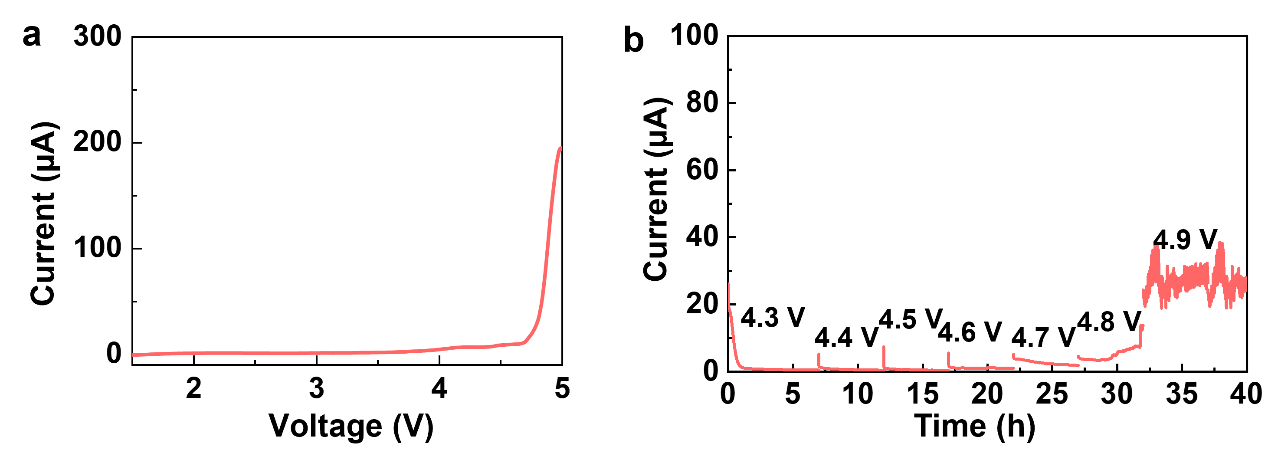


**Figure S11.** The electrochemical stability window (a) and electrochemical floating test (b) in LiFSI/PDOL-30% BFE. Due to the lack of AGG structure in LiFSI/PDOL-30% BFE, the leakage current of this electrolyte is 4.6 V (b).


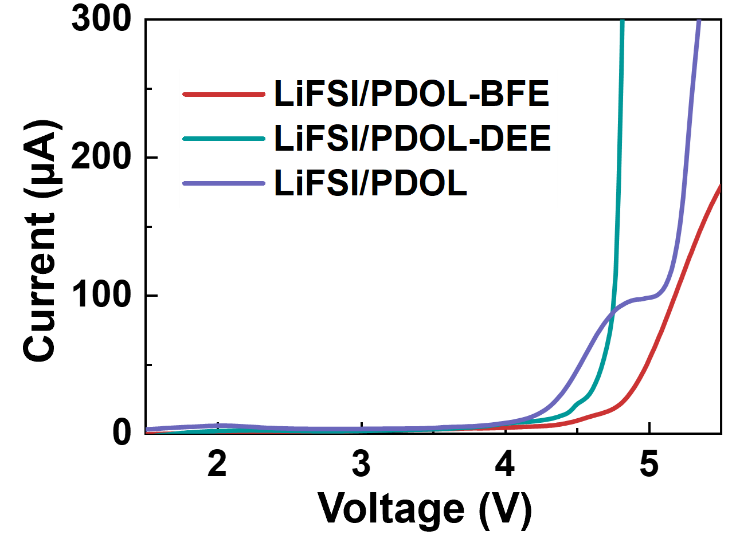


**Figure S12.** The electrochemical stability windows in various electrolytes.


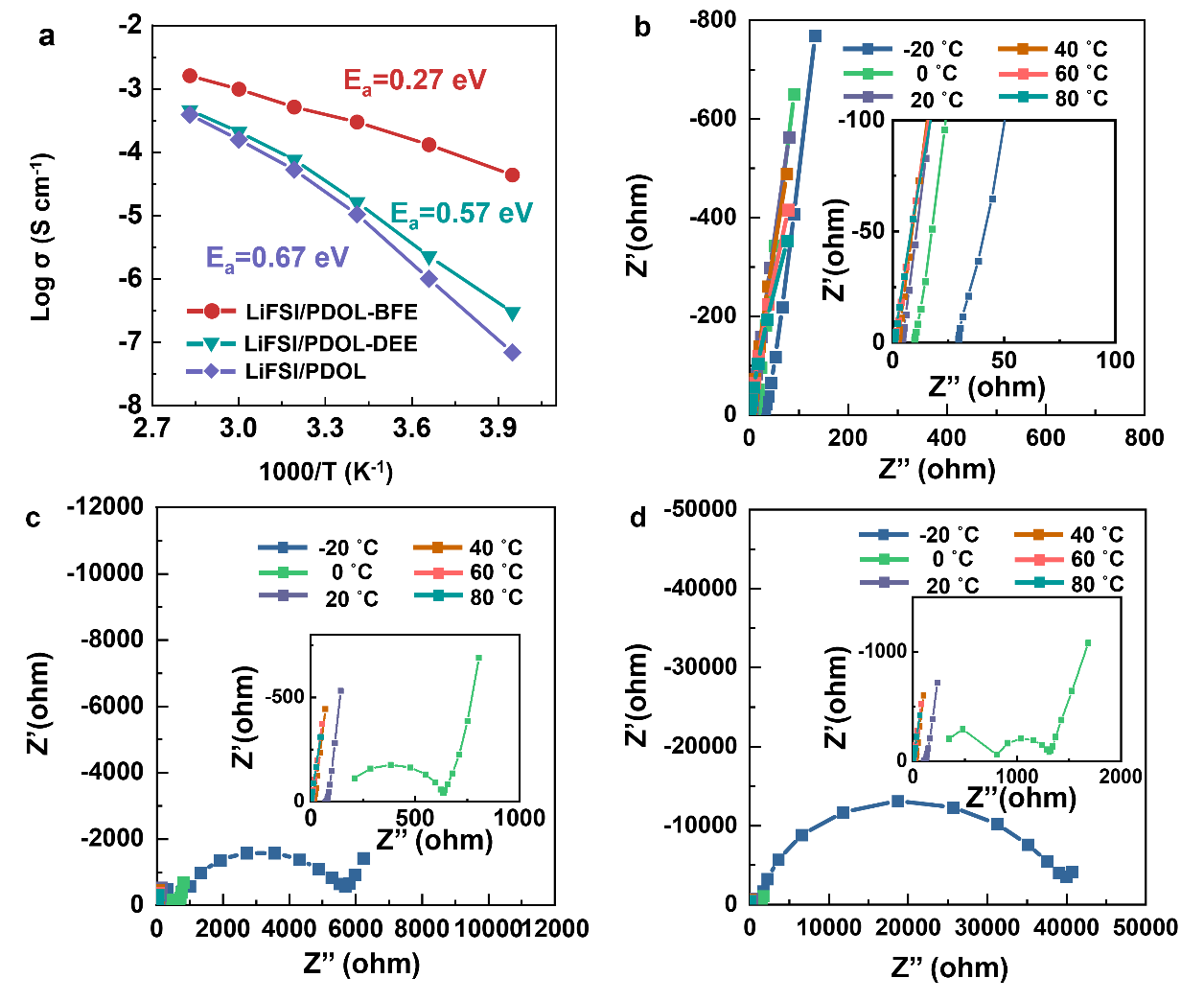


**Figure S13.** The ionic conductivities (a) and Nyquist plots (b) of SS||LiFSI/PDOL-BFE||SS SS||LiFSI/PDOL-DEE||SS and SS||LiFSI/PDOL||SS cells at different temperatures.


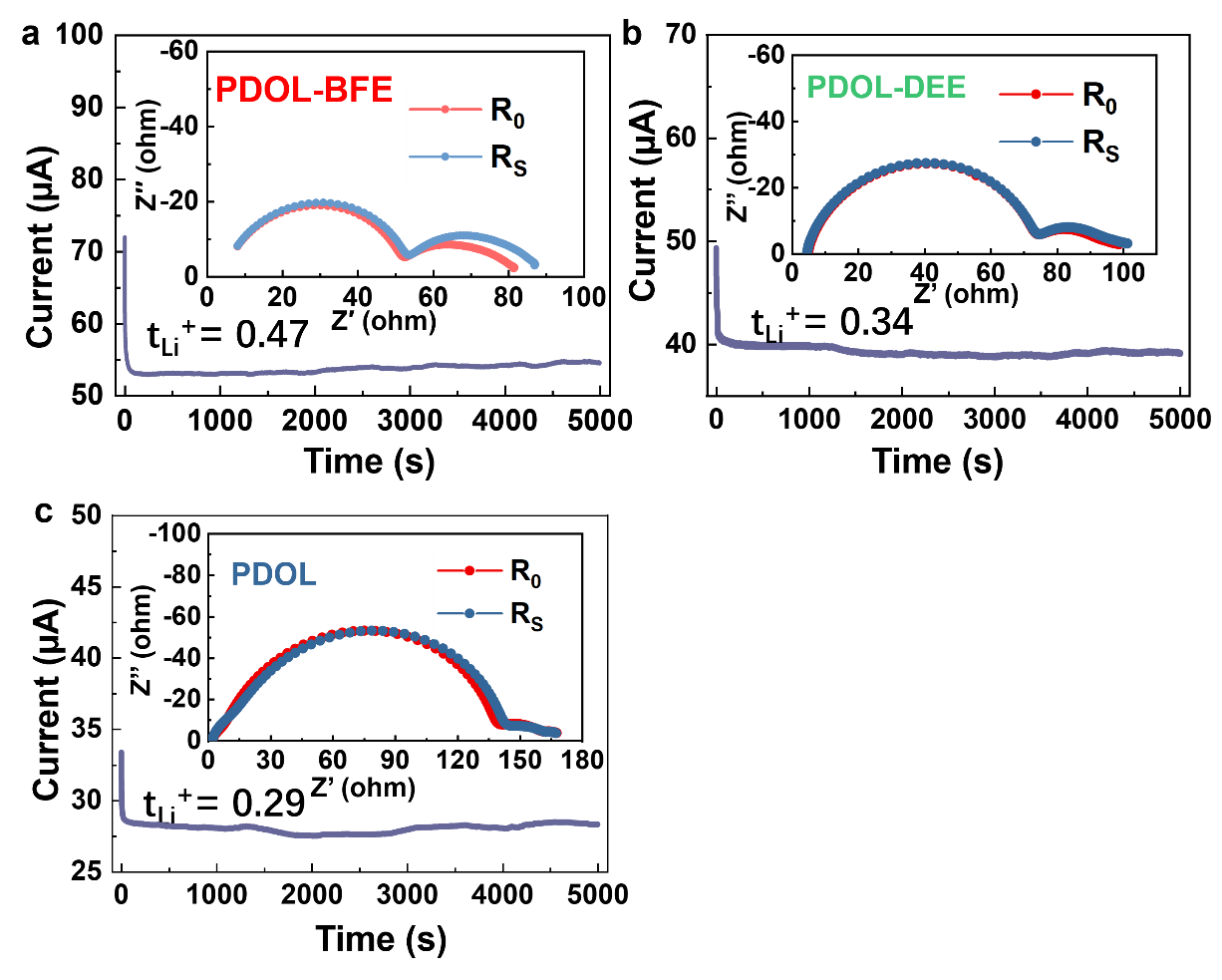


**Figure S14.** The Li^+^ transference number measurements of LiFSI/PDOL-BFE (a), LiFSI/PDOL-DEE (B), and LiFSI/PDOL (c).


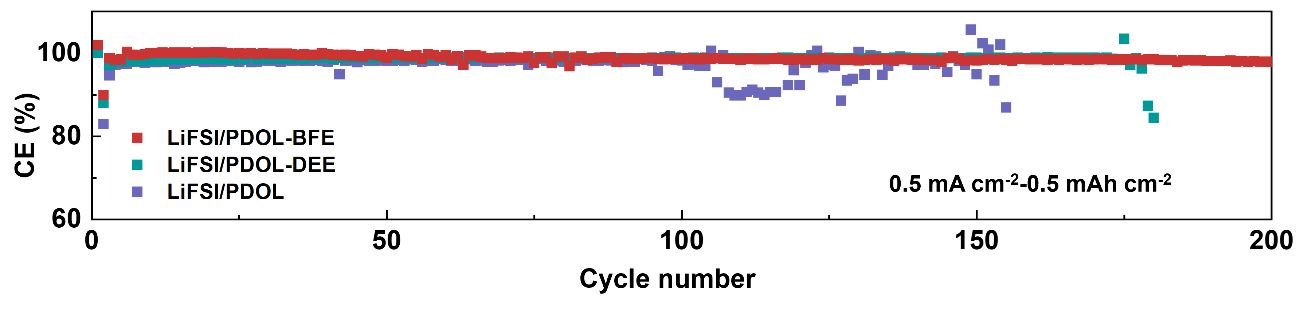


**Figure S15.** Coulombic efficiency of Li||Cu half cells and galvanostatic plating/stripping profiles of Li||Li cells in various electrolytes at 0.5 mA cm^-2^ @ 0.5 mA cm^-2^.


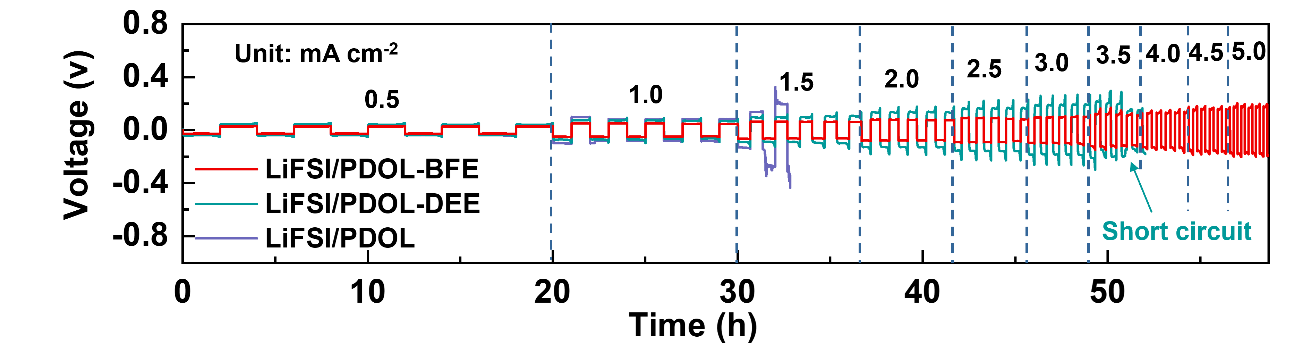


**Figure S16.** Rate performance of Li||Li symmetric coin cells at current densities from 0.5 to 5.0 mAcm^-2^ in different electrolytes.


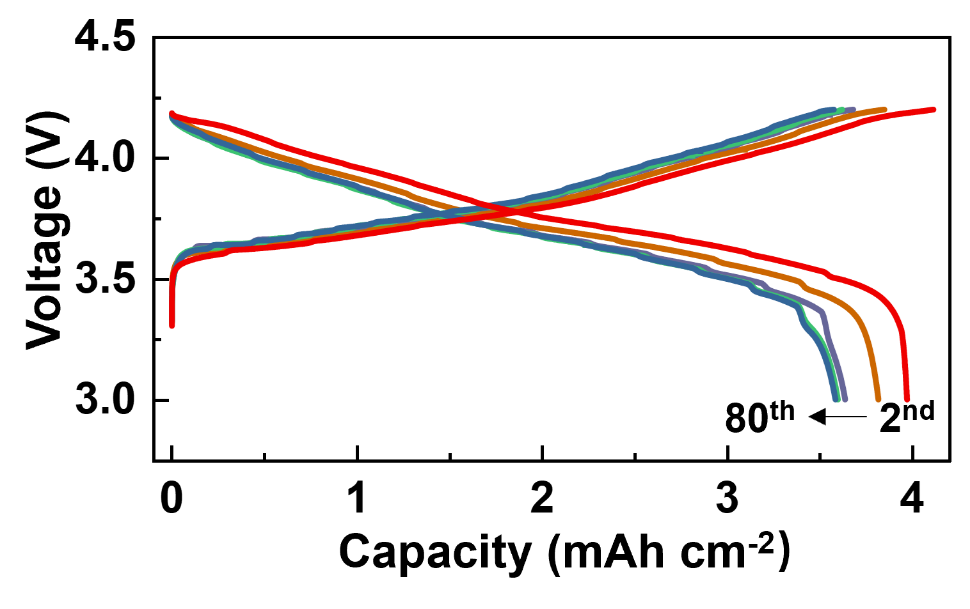


**Figure S17.** The charge/discharge profiles in PDOL-BFE with commercial NCM_811_ cathode.

Table S1

| Component of cell | parameter | value |
| --- | --- | --- |
| Cathode | Areal capacity (mA h cm^-2^) | 3.98 |
|  | Thickness (μm) | 75 |
|  | Mass (mg cm^-2^) | 18.5 |
| Anode | Thickness (μm) | 100 |
|  | Mass (mg cm^-2^) | 9 |
| Electrolyte | Celgard (mg cm^-2^) | 1.0 |
|  | Electrolyte (mg cm^-2^) | 22.99 |
|  | Thickness (μm) | 25 |
| Cell | Total mass (mg cm^-2^) | 51.49 |
|  | Total thickness (μm) | 200 |
|  | Volumetric energy density (Wh L^-1^) | 752.2 |

$$E_{v}=\frac{E}{\sum v_{i}}=\frac{3.78 VХ 3.98 mA h {cm}^{-2}Х 1.0 {cm}^{2}}{200 \mu mХ 1.0 {cm}^{2}}=752.2 Wh L^{-1}$$
